# Crossing the Vacuolar Rubicon: Structural Insights into Effector Protein Trafficking in Apicomplexan Parasites

**DOI:** 10.3390/microorganisms8060865

**Published:** 2020-06-08

**Authors:** Pascal F. Egea

**Affiliations:** Department of Biological Chemistry, David Geffen School of Medicine, University of California Los Angeles, Los Angeles, CA 90095, USA; pegea@mednet.ucla.edu; Tel.: +1-310-983-3515

**Keywords:** apicomplexa, Plasmodium, malaria, Toxoplasma, PTEX, MYR, translocon–protein secretion, parasitophorous vacuole, parasite–host interface, effector, virulence factor, pore-forming membrane protein, AAA+ chaperone, ClpB/HSP104, thioredoxin, protease, anti-parasitic drug

## Abstract

Apicomplexans form a large phylum of parasitic protozoa, including the genera Plasmodium, Toxoplasma, and Cryptosporidium, the causative agents of malaria, toxoplasmosis, and cryptosporidiosis, respectively. They cause diseases not only in humans but also in animals, with dramatic consequences in agriculture. Most apicomplexans are vacuole-dwelling and obligate intracellular parasites; as they invade the host cell, they become encased in a parasitophorous vacuole (PV) derived from the host cellular membrane. This creates a parasite–host interface that acts as a protective barrier but also constitutes an obstacle through which the pathogen must import nutrients, eliminate wastes, and eventually break free upon egress. Completion of the parasitic life cycle requires intense remodeling of the infected host cell. Host cell subversion is mediated by a subset of essential effector parasitic proteins and virulence factors actively trafficked across the PV membrane. In the malaria parasite Plasmodium, a unique and highly specialized ATP-driven vacuolar secretion system, the Plasmodium translocon of exported proteins (PTEX), transports effector proteins across the vacuolar membrane. Its core is composed of the three essential proteins EXP2, PTEX150, and HSP101, and is supplemented by the two auxiliary proteins TRX2 and PTEX88. Many but not all secreted malarial effector proteins contain a vacuolar trafficking signal or Plasmodium export element (PEXEL) that requires processing by an endoplasmic reticulum protease, plasmepsin V, for proper export. Because vacuolar parasitic protein export is essential to parasite survival and virulence, this pathway is a promising target for the development of novel antimalarial therapeutics. This review summarizes the current state of structural and mechanistic knowledge on the Plasmodium parasitic vacuolar secretion and effector trafficking pathway, describing its most salient features and discussing the existing differences and commonalities with the vacuolar effector translocation *MYR* machinery recently described in Toxoplasma and other apicomplexans of significance to medical and veterinary sciences.

## 1. Introduction

Apicomplexa form a large group of parasitic protozoa and are characterized by the presence of complex apical ultra-structures and a unique plastid-like organelle, the apicoplast, product of an ancient endosymbiotic event between a photosynthetic protist (red alga) and a heterotrophic ancestor cell [1,2]. Nearly every vertebrate and a majority of invertebrates can be the host of at least one apicomplexan species. The most notorious members of this phylum include Plasmodium, Toxoplasma, and Cryptosporidium. *Plasmodium falciparum* (*Pf*) and *Toxoplasma gondii* (*Tg*) are the most extensively studied and well-characterized apicomplexans as they are the causative agents of two important human diseases: Malaria and toxoplasmosis, respectively. Cryptosporidium causes cryptosporidiosis, one of the most common water-borne diseases worldwide, and yet it was not identified until 1976; thus, knowledge of its molecular biology is far less advanced.

A vast number of *Plasmodium* species have been identified to infect a broad range of vertebrate hosts, including reptiles, birds, and, mammals (such as humans, monkeys, and rodents). In 2018, Plasmodium was responsible for 219 million malaria cases, claiming 435,000 lives worldwide principally in Asia, sub-Saharan Africa, and South America. Malaria is a mosquito-borne disease, where the parasite undergoes asexual reproduction in the human host and sexual reproduction in the insect. In the human host, *Plasmodium falciparum* first infects hepatocytes, but most of the pathological and clinical manifestations of the disease occur during the blood stage of the infection when it invades and replicates inside red blood cells (RBCs).

It is estimated that an average 15–70% of the world population has been exposed to *Toxoplasma gondiiī,* with infection rates varying greatly from country to country. Toxoplasma infects nearly all warm-blooded animals and while cats are the main reservoir for sexual reproduction, humans are considered as the secondary or intermediate host where asexual reproduction occurs. Infection with Toxoplasma usually produces mild or no observable symptoms. However, in young infants, AIDS patients, and other subjects with weakened immunity, the parasite can cause a fatal illness. Contrary to Plasmodium, Toxoplasma is far more promiscuous as it can infect virtually all types of nucleated cells, although it preferentially invades macrophages to develop a latent/chronic infection.

While members of the same phylum, Plasmodium and Toxoplasma belong to the two distinct orders of Haemospororidia and Eucoccidioridia, respectively. The striking differences in parasitic life cycles, host cell tropism, and the resulting pathologies beautifully illustrate the extreme diversity within this large phylum of eukaryotic pathogens. Yet, despite all this diversity, most apicomplexans are obligate intracellular parasites, and this results in common challenges that all parasites face to successfully invade and thrive in the host cells. In the last two decades, our understanding of the mechanisms underlying apicomplexan pathogenicity and virulence at the level of molecular structures seen at atomic resolution has dramatically expanded. Unfortunately, so far, this large body of knowledge only relates to Plasmodium and Toxoplasma.

## 2. Effector and Virulence Factor Export across the Parasitophorous Vacuole Requires Specialized Vacuolar Translocons

Most apicomplexans are obligate intracellular parasites and dwell in a parasitophorous vacuole (PV) derived from the host cell membrane by invagination. This PV is a protected niche but also represents an additional physical barrier that parasites have to manipulate to thrive in their host and eventually pierce upon egress. In the malaria parasite, the PV membrane (PVM) is a complex parasite–host interface, attached to the parasite plasma membrane (PPM) at distinct contact sites and divided in domains specialized in protein transport or lipid exchange [3,4].

Plasmodium and Toxoplasma extensively remodel their respective host cells via secreted effector proteins, which they introduce during or following invasion [5,6,7,8]. A cornucopia of effector proteins and virulence factors are trafficked into and across the parasitophorous vacuole (PV) to subvert the host cell and successfully mount a cyclic (Plasmodium) or latent/chronic (Toxoplasma) infection. In the last decade, our understanding of this biological process crucial to parasitic life and pathogenesis has greatly improved due to the identification of (1) the molecular complexes mediating translocation across the PVM, and (2) the vacuolar trafficking signals or cues that target these effectors for their secretion across the PVM into the host cell.

**Figure 1:**
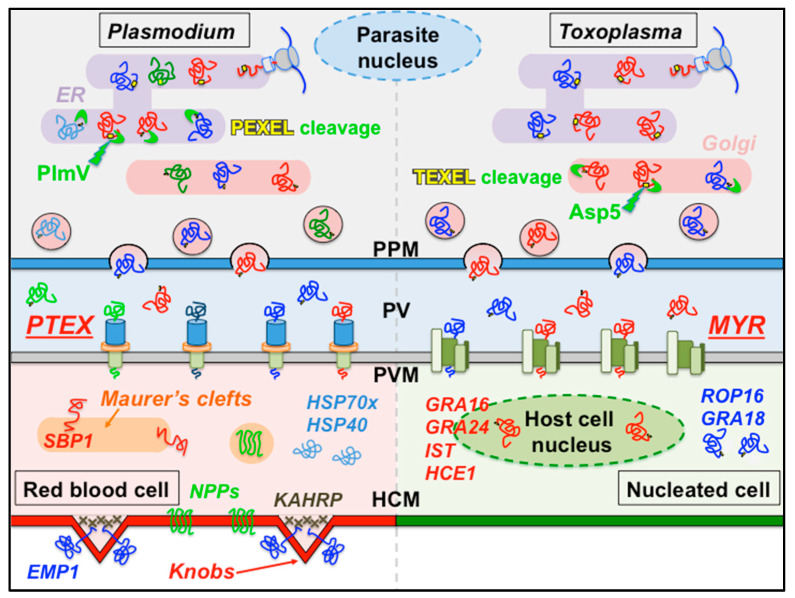
Vacuolar secretion pathways in Plasmodium and Toxoplasma. Effectors and virulence factors follow the general SRP/Sec61-dependent (Signal Recognition Particle/Sec61 translocon) parasitic secretion pathway [18,19] starting in the endoplasmic reticulum (ER) and progressing through the Golgi and vesicular networks. For effectors carrying a vacuolar translocation signal (PEXEL or TEXEL, yellow), a proteolytic licensing step is performed by an aspartic protease, ER-resident PlmV in Plasmodium, or Golgi-resident Asp5 in Toxoplasma. Following vesicular secretion into the PV lumen, two distinct and structurally unrelated vacuolar translocation complexes, *PTEX* in Plasmodium and *MYR* in Toxoplasma, translocate these effectors across the membrane into the infected host cell. Some Plasmodium effectors eventually localize to membranous structures, characteristic of infected red blood cells, such as knobs and Maurer’s clefts. Some Toxoplasma effectors localize to the nucleus of the infected cell. The names of some effector proteins are indicated. PPM, parasite plasma membrane; HCM, host cell membrane.

Two effector translocation systems, the Plasmodium translocon of exported proteins (PTEX) [9] and the MYR protein complex (for host c-*My*c regulation) [10] in Toxoplasma, have been identified. A vacuolar trafficking signal or Plasmodium export element (PEXEL) was identified in some malarial secreted effectors, leading to the definition of a so-called ‘secretome’ or ‘exportome’ of ~463 proteins [11,12,13]. It was then discovered that specific cleavage in the PEXEL motif by plasmepsin V (PlmV), a parasitic ER-resident aspartic acid protease, is required for proper cargo licensing and export [14,15]. In parallel, the existence of a Toxoplasma export element (TEXEL) in a small subset of effectors and the requirement for a functional parasitic Golgi-resident licensing aspartic acid protease (Asp5) to translocate all effectors were established [16,17] (Figure 1).

Plasmodium effector proteins fulfill diverse functions [5]. *Pf*-erythrocyte membrane protein 1 (EMP1) is a highly variable adhesin displayed at the surface of infected RBCs and facilitates attachment to endothelial cells’ surface receptors and uninfected RBCs; it promotes infection and immune response evasion and diminishes infected RBC clearance. EMP1 is localized in knobs, membrane subdomains organized by the knob-associated histidine-rich protein (KAHRP) to remodel the RBC cytoskeleton and surface. Many malarial chaperones (such as HSP70x and HSP40s) are exported beyond the PVM and may assist in effector refolding and RBC remodeling. Some secreted proteins, such as skeletal binding protein 1 (SBP1), participate in the biogenesis and function of Maurer’s clefts, essential parasite-derived membranous structures seemingly involved in some effector protein trafficking and sorting beyond the PVM [20]. Different types of transporters and channels, defining new permeability pathways (NPPs), are secreted and inserted in the RBC membrane and are crucial for the import of small solutes (amino acids, peptides, nucleosides) and inorganic or organic monovalent ions but also the efflux of waste or drugs. They are members of a Plasmodium ‘transportome’, where more than two thirds of its gene products are essential for normal growth in the asexual blood stage [21].

Several Toxoplasma secreted effectors, such as rhoptry (ROP) or dense granule (GRA) proteins, have been identified, including GRA16, GRA18, GRA24, and ROP16 [8]. Dense granule proteins GRA16 and GRA24 interfere with the p53 and MAP kinases signaling pathways, respectively, while IST blocks the interferon response and the ROP16 kinase acts on STAT pathways, resulting in cytokine inhibition [7,22]. More recently, the effector HCE1, an inducer of host cyclin E1, was shown to manipulate host transcriptional responses [23,24].

## 3. The Plasmodium Translocon of Exported Proteins

**Figure 2:**
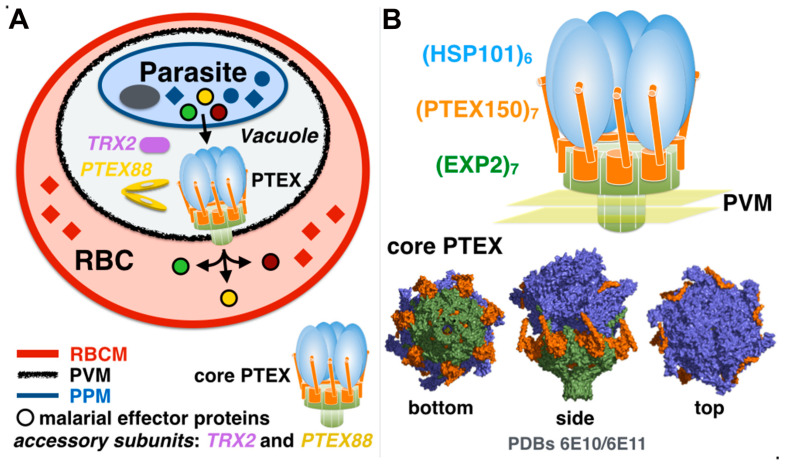
Malaria effector proteins are secreted by PTEX. (**A**) The Plasmodium translocon of exported proteins is a vacuolar membrane protein complex that translocates effector malarial proteins across the parasitophorous vacuole membrane (PVM) surrounding the Plasmodium parasite following invasion of the human red blood cell. (**B**) The core of PTEX is composed of HSP101/ClpB2, PTEX150, and EXP2, is complemented by accessory proteins TRX2 and PTEX88. Simplified drawing of PTEX emphasizing the core subunit stoichiometry and the six-to-seven symmetry mismatch, with three views of the core PTEX structure determined by cryo-EM [26].

In their seminal work, De Koning-Ward et al. characterized the first apicomplexan vacuolar translocon, PTEX, as a large PVM-associated complex composed of five subunits: EXP2, PTEX150, HSP101, thioredoxin-2 (TRX2), and PTEX88 [9]. The structure of the stable detergent-resistant EXP2/PTXE150/HSP101 ternary core complex [25], purified from parasite-infected red blood cells, was solved at near-atomic resolution by cryo-electron microscopy (cryo-EM) [26,27,28] (Figure 2 and Table 1). The three core subunits are essential to parasite survival [29,30,31,32].

The structure revealed the subunit structures together with the complex stoichiometry and its intricate assembly. Furthermore, the endogenously purified PTEX complexes extracted from parasites were caught in the act, trapped with endogenous parasitic cargo. This provided some mechanistic insights into effector translocation across the PVM by this unique translocon.

### 3.1. Exported Protein-2: An Unusual Pore-Forming Protein with Multiple Functions?

**Figure 3:**
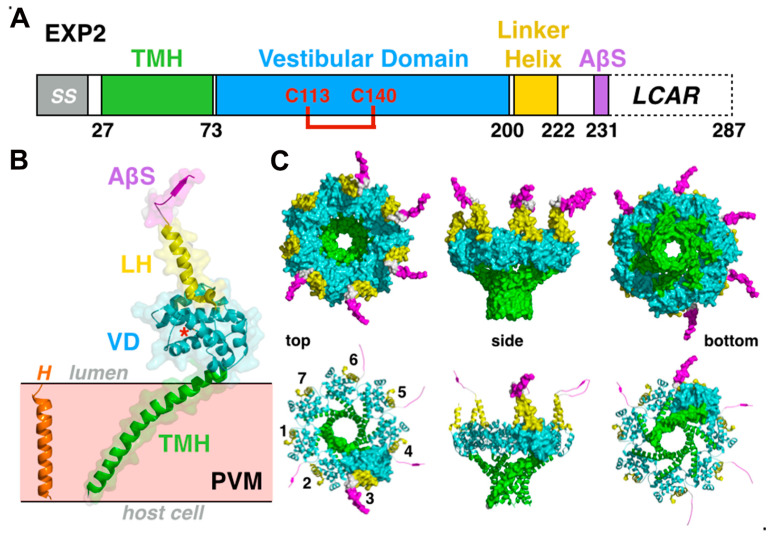
The protein-conducting pore-forming protein EXP2. (**A**) Schematic organization of EXP2 including: SS, signal sequence; TMH, transmembrane helix; VD, vestibular domain; LH, linker helix; AβS, assembly β-strand; LCAR, low-complexity acidic region and the strictly conserved disulfide bond between residues C113 and C140. (**B**) EXP2 protomer structure in transparent surface representation. The red asterisk marks the position of the strictly conserved disulfide bond. A canonical TMH perpendicular (H) to the plane of the membrane is shown as a comparison with the highly tilted and longer TMH of EXP2. (**C**) Heptameric arrangement of the EXP2 transmembrane protein-conducting pore shown from the top (vacuolar lumen), side (membrane plane), and bottom (host cell cytoplasm) orientations.

Exported protein-2 (gene PF3D7_1471100) is highly conserved across the different *Plasmodium* species. Variability occurs mostly at the *C*-terminus of the protein harboring sequences of variable length and enriched in aspartic or glutamic acid. In the cryo-EM structure, this *C*-terminal region (~40 residues) could not be resolved, suggesting it is flexible and/or disordered (Figure 3 and Figure S1). The EXP2 protomer can be divided into a trans-membrane helix (TMH, residues G27-R73), a solvent-accessible vestibular domain (residues Y73-L197) exposed in the vacuolar lumen stabilized by a conserved disulfide bond (C113–C140), and a rigid linker helix (P197-K221) followed an assembly β-strand (residues G226-S235). Embedded in the PVM, seven protomers of EXP2 associate to form a funnel-shaped pore, acting as the membrane protein-conducting channel (Figure 3). The TMH consists of a single 45-residue-long continuous α-helix that crosses the ~38Å-thick lipid bilayer at an unusually steep ~45° angle. Biochemical studies have since validated the structure, revealing that the *N*-terminus of EXP2 forms the membrane-associated pore [33]. The transmembrane parts of the EXP2 heptamer generate a pore of about ~20Å in diameter capable of accommodating a folded α-helical segment (~12Å in diameter) while the luminal vacuolar domains assemble in a funnel-shaped cavity with a much wider diameter of ~43Å (Figure S3). Although secondary structure prediction algorithms predicted that EXP2 was essentially α-helical, none of the TMH prediction algorithms detected the presence of its transmembrane helix. This can be explained upon examination of the structures of the protomer and its heptameric assembly into the functional protein-conducting pore. The membrane-spanning helix is amphipathic with a strongly hydrophobic face interacting with the membrane lipids and, quite remarkably, a hydrophilic/polar face constituting the lumen of the pore.

EXP2 also serves as a small molecule/solute-permeable vacuolar channel [31] and is functionally equivalent to *Toxoplasma gondii* dense granule proteins GRA17 and GRA23 [34,35]. Two vacuolar separate molecular pools of EXP2 might thus coexist: One consisting of EXP2 assembled in the PTEX complex and one pool of ‘free’ EXP2 (without PTEX150 and HSP101), possibly associated with another vacuolar membrane protein, exported protein-1 (EXP1) [36], essential to the maintenance of the vacuole ultra-structure and EXP2 organization and function [37]. If EXP2 also exists under other functional form(s), does it adopt the same structure(s) and oligomeric state as in the context of PTEX? This would be important in regard to the structure, size, and selectivity of the pore. Understanding the mechanisms of vacuolar solute permeability will necessitate further structural characterization of the GRA17 and GRA23 proteins in Toxoplasma or of the Plasmodium EXP2 protein involved in the activity distinct from its protein translocation function as a core subunit of the PTEX.

### 3.2. Disorderly Functional: The Adaptor Protein PTEX150 and the Roles of Low-Complexity Regions in the Plasmodium Proteome

**Figure 4:**
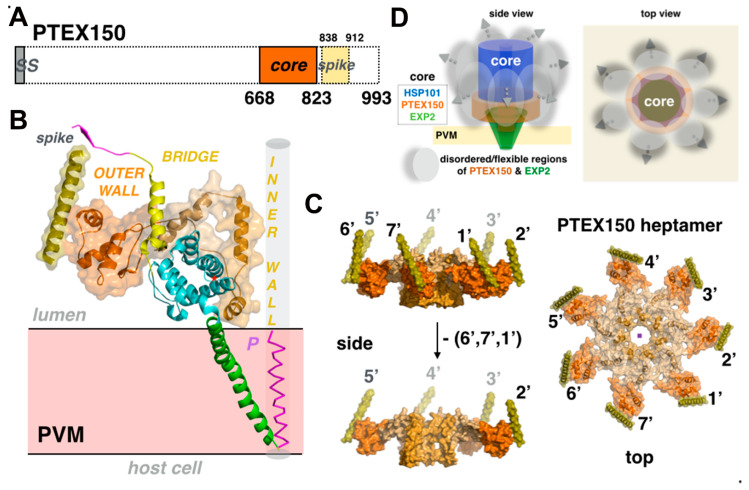
The adaptor protein PTEX150 and disordered regions in Plasmodium proteome. (**A**) Schematic organization of PTEX150, including SS, signal sequence. Only ~18% of the total protein, corresponding to the core and spike domains, are resolved in the structure. (**B**) The PTEX150 protomer structure in transparent surface representation. The core of one PTEX150 protomer can be divided into an outer and inner ‘wall’ separated by a bridge and sits on top of the vestibular domain of the EXP2 protomer (colored as in Figure 3). The hydrophilic inner wall contributes to the lining of the central pore. A model helix (P) indicates the axis of the central pore along the protein-conducting path (transparent grey cylinder). (**C**) Heptameric arrangement of PTEX150. (**D**) Schematic showing the overall contribution of the disordered/flexible protein regions in PTEX.

The core subunit PTEX150 (gene PF3D7_1436300) is a 993-residue-long soluble protein with no known homologues found upon survey of the protein sequence or protein structure databases. Because of this lack of any known functional domains, the exact role of PTEX150 was unknown until the PTEX structure revealed that it functions by tethering the soluble ATPase HSP101 to the membrane-embedded EXP2 but also by completing and most likely stabilizing the protein-conducting pore in association with the transmembrane channel EXP2 (Figure 3 and Figure 4). Remarkably, only residues S668-D823 of PTEX150 could be assigned and modeled unambiguously in the density maps, thus revealing that nearly ~82% of the protein is disordered and/or so dynamic that it was averaged out during three-dimensional reconstruction (Figure S2).

In the cryo-EM structure, seven protomers of PTEX150 interact with seven protomers of EXP2 to form the intimately intertwined rigid tetradecameric channel assembly. The vestibular cavity in the EXP2 heptamer is filled up by the seven inner-wall domains of the PTEX150 heptamer; as a result, the pore geometry is no longer funnel shaped but roughly more cylindrical, with an average diameter of ~15–20Å (Figure S3). The structured core of PTEX150 is strongly although not perfectly conserved within all *Plasmodium* species (Figure S2). Two additional protein segments, presumably belonging to PTEX150 and corresponding to spike-like and claw-like structures projecting towards the ATPase HSP101, were also identified, but their exact sequence could not be assigned. This correlates well with the prediction of disordered regions and also the presence of 17% of asparagine and 13% of aspartate nearly all present in the disordered regions of PTEX150 and therefore not observed in the structure. Of the three core PTEX components, only EXP2 and PTEX150, but not its essential ATPase HSP101, contain acidic (aspartate/glutamate) or asparagine-rich repeats, with PTEX150 being the most extreme case (30% of Asn/Asp and 10% of Glu). Accessory subunit PTEX88 also contains asparagine repeats (13% of Asn) (Table 2). Within the PTEX core, these low-complexity regions are contributed exclusively by PTEX150 and EXP2, and account for an astonishing 43% of its total mass (~0.68 MDa out of 1.59 MDa); they are exposed on the outer surface of the complex (i.e., not towards the substrate translocation and protein-conducting paths) and are predicted as disordered (Figure 4D).

With a 70% AT-rich genome, Plasmodium’s genome exhibits one of the highest codon biases in all eukaryotic genomes. As a result, nearly 25% of the proteome contains asparagine or aspartate expansions. The functional significance and importance of these asparagine/aspartate-rich regions in Plasmodia is not well understood. One hypothesis proposes that these flexible disordered regions might act as entropic bristles, enhancing the solubility of proteins and complexes [38,39,40]. A study of the ER-to-Golgi tether OSBP-related protein 4 suggests that low-complexity sequences could form an entropic barrier that restrains protein orientation, limits protein density, and facilitates protein mobility (in-plane lateral diffusion) in the crowded environment of membrane contact sites [41] by analogy with the narrow and crowded PV lumen. This, however, contrasts with functional studies showing that the *Plasmodium falciparum* asparagine-rich proteome is more prone to aggregation [42,43]; similar observations were made in the amoeba *Dictyostelium discoideum,* another unrelated eukaryote with a proteome enriched in glutamine/asparagine repeats [44].

The genes encoding the three core PTEX subunits are on three distinct chromosomes. Close examination of the EXP2/PTEX150 tetradecamer structure shows that one monomer of PTEX150 contacts not one but three adjacent EXP2 protomers. This remarkable and exquisite intricacy raises several questions regarding PTEX biogenesis and may be cooperative assembly in vivo and the intrinsic stability of EXP2 and PTEX150 (Figure S3). Proteins PTEX150 and HSP101 are synthesized at the schizont stage while EXP2 expresses at earlier stages. Before invasion of the red blood cell, these components are stored within small apical organelles of the invasive merozoites, the dense granules [25]. Upon cell entry, PTEX is released and inserted in the PVM; interestingly, the PTEX pool undergoes little turnover for most of the remainder of the parasite development.

### 3.3. Transmembrane Pore Rigidity, Geometry, and Physicochemical Properties

Cyclic or circular seven-fold symmetry (C7) is not uncommon in proteins whether they are soluble or membrane embedded; for membrane proteins, the best-described cases of C7 symmetrical assemblies belong to a large family of pore-forming toxins (PFTs) [45] divided in helical (α-PFTs) and β-sheet pore-forming toxins (β-PFTs) that include the C7-symmetric staphylococcal α-hemolysin [46] and anthrax protective antigen pore [47] (Supplementary Figure S4). However, besides EXP2, they have so far been no examples of C7 symmetrical membrane proteins using a single helix as the transmembrane-spanning segment. The lumen of the rigid PTEX150/EXP2 pore is polar/hydrophilic and wide enough to accommodate a fully folded α-helical segment. This raises the question as to whether some spontaneous refolding of the cargo already takes place inside the transmembrane section of the channel before they enter the cytoplasm of the red blood cell. While PTEX clearly displays translocase activity ensuring the secretion of hundreds of effector proteins across the PVM, there is no direct evidence that it can also function as an “insertase”, capable of chaperoning hydrophobic transmembrane domains of integral vacuolar membrane proteins into the lateral plane of the PVM [48]. In comparison, the main subunit of the heterotrimeric Sec61/SecY universal translocon forms an hourglass-shaped protein-conducting channel, capable of accommodating unfolded polypeptides for their secretion across the membrane but also suited for the partitioning of folded transmembrane helical segments for their lateral insertion into the bilayer [49,50]. Sec61/SecY has dual translocase/insertase activities (Figure S4). Sec61/SecY is a remarkably plastic and malleable structure [51] capable of maintaining a seal to small solutes and ions while translocating polypeptides across the membrane [52] thanks to a ring of hydrophobic residues and a plug helix located in the middle of its hourglass-shaped pore. By comparison with the anthrax pore that threads unfolded polypeptides and the Sec61/SecY channel capable of translocating both unfolded and partially folded proteins, the PTEX150/EXP2 pore lacks any seal-, plug-, or clamp-like structural elements to prevent the free diffusion of solutes (water, ions) or small organic molecules and act as a gate.

### 3.4. Energizing Effector Translocation in Plasmodium: The AAA+ Protein Unfoldase HSP101

#### 3.4.1. HSP101/ClpB2: The AAA+ Protein Unfoldase that Drives Protein Export

**Figure 5:**
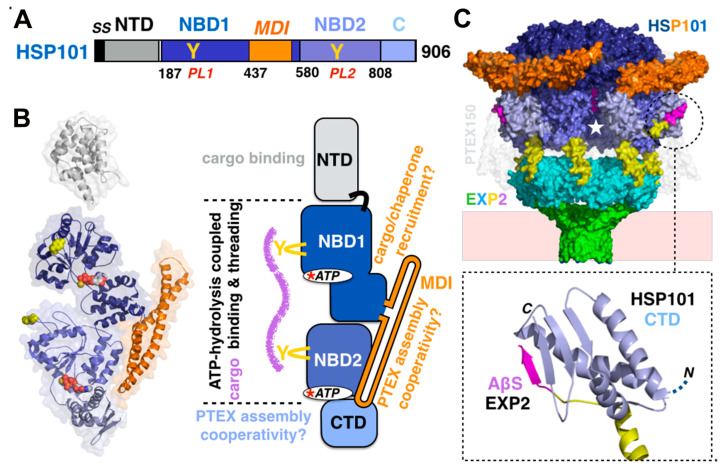
The hexameric AAA+ protein unfoldase HSP101/ClpB2 and its interactions with the PTEX150/EXP2 tetradecamer. (**A**) Domain organization of HSP101/ClpB2. (**B**) Structure of the HSP101 protomer with domains colored as in (**A**), tyrosine pore loops (yellow) and ATP molecules (atom spheres). The cryo-EM structure of the HSP101 lacks the *N*-terminal domain whose crystal structure was solved separately [60]. The schematic indicates the activities and functions of each domain. (**C**) Six-to-seven symmetry mismatched assembly of the hexameric HSP101 on the C7 symmetric PTEX150/EXP2 tetradecamer PTEX150 is rendered in transparent light grey to emphasize the direct interactions between HSP101 and EXP2. Endogenous cargo (magenta spheres) is present in the HSP101 central translocation pore (white star). The inset details the β-augmentation anchoring the CTD of HSP101 to the *C*-terminal assembly β-strand of EXP2 (colored as in Figure 3).

Most proteins are thermodynamically stable in their folded state and translocons thus require energy sources to drive translocation. Several sources of energy, such as the binding and hydrolysis of ATP (or GTP) [53] or membrane potential (proton gradients) [54,55,56], are used to drive translocation. In PTEX, substrate unfolding and threading across the membrane are performed and driven by the HSP101 (gene PF3D7_1116800) subunit, a chaperone belonging to the AAA+ (ATPase associated with diverse cellular activities) protein superfamily. HSP101 is capable of converting ATP-driven conformational changes into mechanical forces sufficient to unfold and translocate polypeptides. Based on its ability to couple ATP-binding and hydrolysis to changes in the folding and/or assembly states of its substrate proteins, HSP101 belongs to the HSP100 class of proteins [57]. In terms of nomenclature, HSP101 is a class I AAA+ protein and shares a conserved domain organization with other members of the HSP100 subgroup, such as the protein disaggregases HSP104 and ClpB [58] characterized by the presence of two active nucleotide binding domains (NBD1 and NBD2) constituting the catalytic core. This catalytic core is flanked with an *N*-terminal domain (NTD) and a *C*-terminal domain (CTD). The NBD1 contains a middle domain insertion (MDI) characteristic of all ClpB/HSP104; based on the presence of specific protein motifs, *Pf*-HSP101’s accurate nomenclature name is *Pf*-ClpB2 [59]. *Pf-*HSP101 NBDs share 40% and 39% sequence identity with the NBDs of *Escherichia coli (Ec)* ClpB and *Saccharomyces cerevisiae (Sc)* HSP104, respectively. Each NBD harbors characteristics of Walker A and B motifs involved in ATP binding and hydrolysis (Figure 5 and Figure S5).

Hexameric HSP101 assembles on top of the PTEX150/EXP2 tetradecamer; this results in a continuous translocation path, shielded from the solvent, that runs from the apex of the ATPase through PTEX150 and EXP2 to the exit on the other side of the PVM. Although PTEX150 plays the role of an adaptor between the HSP101 and EXP2 channel, a direct and essential non-covalent association between the six *C*-terminal domains of HSP101 and five of the seven assembly β-strands at EXP2 *C*-termini firmly anchors the ATPase to the transmembrane EXP2 pore [26]. This interaction constitutes an example of β-addition [61,62] and more specifically β-augmentation, where the *C*-terminal assembly β-strand of EXP2 associates with the *C*-terminal β strand of the CTD of HSP101 to expand its three-stranded β sheet into a four-stranded β-sheet. Knockdown of EXP2 is lethal to the parasite [61,62]. While this defect can be rescued by a version of EXP2 lacking the non-conserved *C*-terminal acidic region (LCAR in Figure 3 and Figure S1), such complementation fails when the assembly β-strand is also lacking. This interaction is thus critical for PTEX assembly and function [26]. In EXP2, the main role of the linker helix is to project the *C*-terminal assembly strands upwards, over the protein layer contributed by PTEX150, towards HSP101 CTDs and enable the direct interaction between EXP2 and HSP101 by β-augmentation. However, as each linker helix closely interacts with the α-helix present in the bridge element of each PTEX150 protomer (Figure 4A), it is likely to also play the subtler role of a sensor, coupling HSP101/EXP2 to PTEX150.

Six-to-seven symmetry mismatch is not unprecedented in protein assemblies [63]. The bacterial ClpAP is an ATPase-dependent chaperone-assisted protease, consisting of a proteolytic component ClpP and the AAA+ chaperone ClpA [64]. This symmetry mismatch likely provides the necessary flexibility to the ATPase to undergo large conformational changes during the cycles of cargo unfolding and threading coupled to ATP-binding and hydrolysis while remaining tethered to the rigid membrane-associated PTEX150/EXP2 protein-conducting assembly.

#### 3.4.2. Hexameric Spiral Staircase Assembly and Cargo Translocation Coupled to ATP-Binding and Hydrolysis

**Figure 6:**
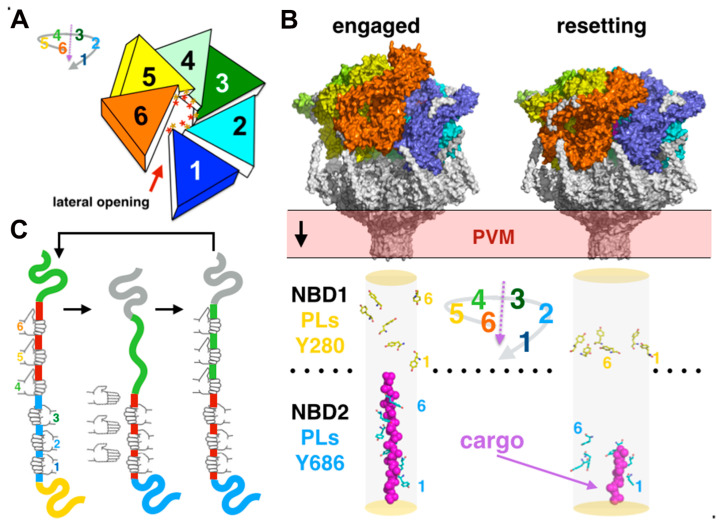
Spiral staircase hexameric assembly and substrate translocation in HSP101. (**A**) Schematic of the splayed spiral staircase assembly of HSP101. Each ATPase monomer is colored differently. (**B**) The two conformational states of PTEX resolved by cryo-EM with the conformations adopted by each of the twelve conserved pore loop tyrosines; the observed unfolded cargo is shown as magenta spheres. (**C**) Schematic representation of a cargo threading cycle in the NBD2 of HSP101. Each hand represents a pore loop in the NBD2. The ‘active hand’ pore loops 4–6 move up and down the ATPase translocation pore and thread it to the ‘static hand’ pore loops 1–3.

The PTEX complexes solved by Ho and Beck were purified directly from parasites and contained trapped unfolded parasitic cargo proteins engaged in the HSP101 ATPase translocation channel. Two distinct conformational states could be reconstructed (‘engaged’ versus ‘resetting’ states). The hexameric ATPase shares a pseudo-helical arrangement that resembles a splayed spiral staircase observed in many AAA+ proteins [65,66] (Figure 6). The ATPase domains encircle the translocating substrate along a relatively narrow central pore (~10 to 20 Å diameter). The unfolded substrate adopts an extended β-strand conformation, with its side chains pointing at the pore loops, also arranged into a spiral. The conserved pore loop tyrosines of the NBD2 intercalate along the substrate polypeptide backbone, maintaining the elongated unfolded conformation.

Both states were trapped in the presence of ATPγS, a slowly hydrolysable ATP analog that mimics the ATP-bound ground state. Upon comparison of these states, a simple model for cargo threading could be derived. An ‘active hand’ contributed by the three apical NBD2 pore loops grab the polypeptide and actively feeds it through the ‘passive hand’ contributed by the three basal NBD2 pore loops by moving downward along the translocation path. Upon threading and release, the system resets and as the active hand returns to the upward position it engages a new upstream portion of the polypeptide substrate. Interestingly and quite surprisingly in light of the other cargo-bound HSP104/ClpB structures available, in both states, a resolved no cargo density can be observed in the apical NBD1s or in the PTEX150/EXP2 section of the complex; thus, many mechanistic aspects of translocation remain unknown.

#### 3.4.3. The Role of *N*-Terminal Domains in Cargo Unfolding, Binding, and Recognition

Export across the PVM by PTEX requires unfolding of the substrates [67,68], and while the HSP101 ATPase threads unfolded cargo through the membrane, prior unfolding occurs at the parasitic plasma membrane [69]. The exact function of the NTD of HSP100s from the ClpB subgroup has remained elusive; the current model proposes that ClpB recognizes exposed hydrophobic stretches in unfolded or aggregated client proteins via a substrate-binding groove in its NTD. In contrast, the *N*-terminal domains of chaperones from the ClpA and ClpC subgroups have been shown to specifically interact with adaptor proteins, such as ClpS [70] also present in Plasmodium [71] or MecA [72], respectively, to regulate the delivery of substrate/client proteins destined for proteolytic degradation by a ClpP protease [73] following their unfolding by the ATPase; the NTD of bacterial ClpC has also been shown to directly recognize the phosphorylated arginine residues in proteins targeted for degradation by ClpP [74]. ClpB/HSP104 proteins rescue damaged proteins from toxic aggregates but do not partner with any proteases while ClpA and ClpC function as regulatory components of ATP-dependent protease complexes [57].

**Figure 7:**
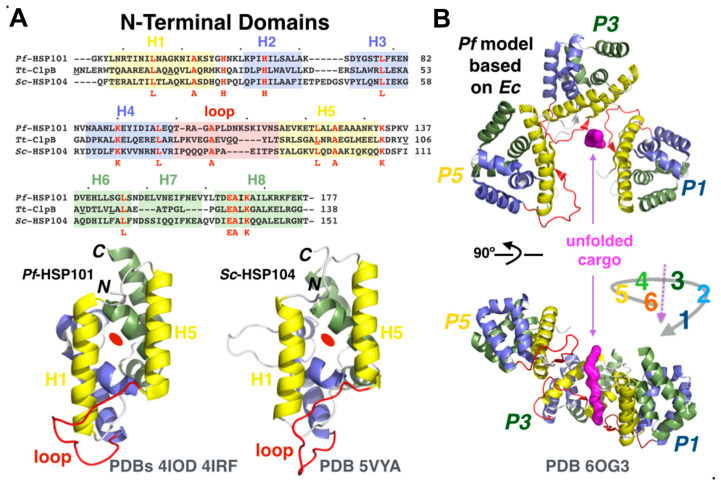
Promiscuous substrate binding by the *N*-terminal domain of HSP101. (**A**) Structure-based sequence alignment of the NTDs from *Pf*-HSP101, *Tt*-ClpB, and *Sc*-HSP104. Residues from *Tt*-ClpB NTD shown to interact with denatured proteins based on CSP NMR analysis [75] were located in helices H4 and H5 and the connecting loop. (**B**) Possible binding mode for denatured substrates by a trimer of *N*-terminal domains of *Pf*-HSP101 in the PTEX based on the structure of *Ec*-ClpB bound to casein [76]. As in *Ec*-ClpB NTDs, *Pf*-HSP101 NTDs could engage denatured extended polypeptides using a hydrophobic groove delineated by helices H1 and H5 (yellow) and the loop (red) between helices H4 and H5 [60].

In chemical shift perturbation (CSP) NMR experiments, the largest CSPs were observed for NTD residues M1–A17 (H1), G80–L91 (loop between H3–H4, H4), V106–V108 (loop between H4–H5), and L111 (H5) in *Thermus thermophilus* (*Tt*) ClpB [75]. When mapped on the high-resolution crystal structures of *Pf*-HSP101/ClpB2 *N*-terminal domains, this corresponds to a hydrophobic batch capable of binding a range of unfolded peptidic sequences [60] (Figure 7A). Furthermore, the NTD of *Tt*-ClpB has regulatory roles that include blocking the translocation channel in the absence of substrate and destabilizing client proteins upon binding, thus priming them for subsequent unfolding and disaggregation [75]. *Pf-*HSP101 and *Tt*-ClpB NTD share 20% sequence identity with a root-mean square deviation of 2.4Å; on the other hand, *Pf*-HSP101 and *Sc*-HSP104 NTDs share 24% sequence identity with a root-mean square deviation of 2.1Å.

The *N*-terminal domain of Plasmodium HSP101/ClpB2 was not resolved in the cryo-EM structure [26], indicating that the domains were adopting different positions relative to the remaining hexameric ATPase. This contrasts with the yeast HSP104 cryo-EM structures, where the six *N*-terminal domains of the ATPase could be resolved in the AMPPNP- [77,78] and ADP- [78] bound states and more recently in the case of the ATPγS-bound state of the bacterial ClpB from *E. coli* [76], where a trimer of *N*-terminal domains defines the entrance channel binding the polypeptide substrate at the topmost upper part of the hexameric spiral-shaped assembly of NBDs. Such a difference could also be explained by the mode of preparation of the different AAA+ chaperone/substrate complexes used for structure determination. For PTEX, Ho and Beck used endogenous complex, directly purified from parasites and serendipitously engaged with diverse endogenous substrate proteins trapped in the presence of ATPγS. Cargo-bound yeast HSP104 [78] and bacterial ClpB [76,79] structures were obtained using purified recombinant proteins trapped with denatured casein, a relatively well-defined purified protein substrate. Nevertheless, the high-resolution crystal structure of the *N*-terminal domain of HSP101/ClpB2 was solved, revealing a hydrophobic surface capable of binding exposed stretches of hydrophobic residues [60] and providing a complete structural coverage of the malarial ATPase. Assuming that plasmodial HSP101 shares some commonalities with yeast HSP104 and bacterial ClpB, a similar model can be proposed for the HSP101–unfolded cargo interaction (Figure 7B).

### 3.5. Accessory Proteins TRX2 and PTEX88

Two other auxiliary subunits, thioredoxin-2 (gene PF3D7_1345100) and PTEX88 (gene PF3D7_1105600), were also characterized as being functionally and physically associated with the core PTEX components [9,25,80,81] albeit at sub-stoichiometric proportions. While subunits EXP2, HSP101, and PTEX150 are absolutely essential to PTEX-mediated protein trafficking and to parasite survival in the human host [29,30,31,32], this is not the case for auxiliary proteins TRX2 and PTEX88. The TRX2 gene can be deleted in Plasmodium, and TRX2 knock-out parasites display reduced growth rates in vivo and reduced capacity to cause the most severe forms of the disease [82]. Thus, it is proposed that while not essential to parasite survival, TRX2 may improve or facilitate the activity of PTEX. The situation is more complex and somehow confusing for PTEX88. Although PTEX88-deficient parasites are less virulent and have delayed growth in vivo, they do not display a clear protein export defect phenotype [83,84].

**Figure 8:**
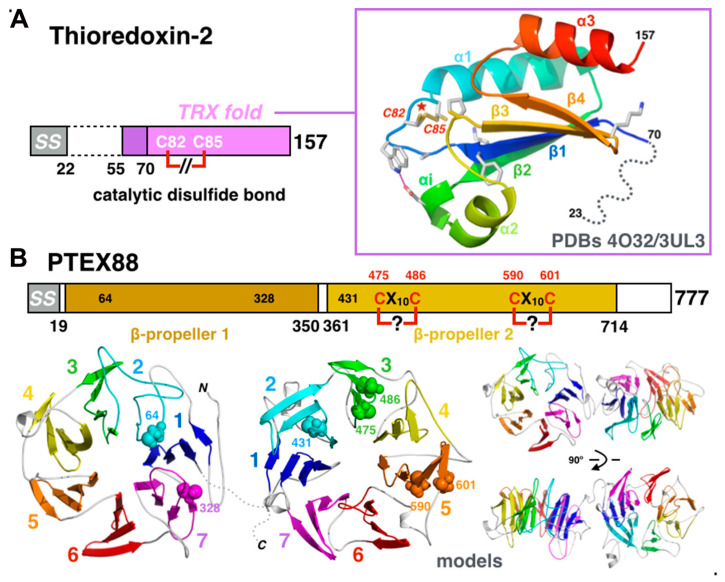
Accessory proteins TRX2 and PTEX88. (**A**) Crystal structure of the Plasmodium thioredoxin-2. The C_82_XXC_85_ catalytic dyad residues (marked with a star) are shown in the reduced and oxidized forms simultaneously observed in the crystal [86]. (**B**) Homology model of the Plasmodium protein PTEX88. The homology model of PTEX88 suggests the presence of two seven-bladed β-propeller domains shown, with each blade colored using a rainbow pattern with a disulfide bond in the predicted C_590_X_10_C_601_ motif of the *C*-terminal propeller. The unknown relative orientation between the two propellers domains is thought to be important in defining the functional protein interfaces.

High-resolution crystal structures of the thioredoxin-2 (TRX2) [85,86] revealed that the 157-residue-long malarial protein contains a canonical βαβαββα thioredoxin domain characterized by a labile disulfide bond, formed by the canonical CX_2_C catalytic dyad observed in protein-disulfide reductase/oxidase enzymes [87,88] and that acts as the electron donor to reduce disulfide bonds in client proteins (Figure 8A). Export across the PVM by PTEX requires unfolding of the substrates [67,68]; since the presence of disulfide bonds can block export, the genuine protein-disulfide reductase/oxidase Plasmodium TRX2 might relieve this type of blockade and thus facilitate PTEX activity by reducing disulfide bonds or preventing their formation in cargo proteins destined for export across the parasitophorous vacuolar membrane.

PTEX88 is a 777-residue-long protein. The presence of a signal sequence indicates that it is secreted into the vacuolar lumen. Like PTEX150, no homologue has been found throughout the sequence databases. No structural information is available for PTEX88 so far. PTEX88 was shown to be more closely associated, albeit in a more transient and dynamic way, with HSP101 [81] and the exported protein-interacting complex (EPIC) [89], another PVM complex involved in the trafficking of virulence determinants, such as EMP1. Based on these findings, PTEX88 might help in the recognition and delivery of subsets of exported proteins to the HSP101 ATPase. Homology modeling in *I-TASSER* [90] suggests that it might be composed of two consecutive so-called seven-bladed β-propeller domains separated by a short linker (Figure 8B). β-propellers are symmetrical structures made of 4 to 10 repeats of a four-stranded antiparallel β-sheet motif depicted as a blade. All β-propeller proteins adopt disc-like shapes with a central channel of the diameter increasing with the number of blades; these proteins are involved in a diverse set of functions [91]. In PTEX88, the predicted *C*-terminal propeller contains five of the seven cysteines present in the whole protein and two potential CX_10_C motifs can be identified between the C475/C486 and the C590/C601 pairs. However, examination of the homology model suggests that only cysteines C590 and C601 are close enough to potentially engage in disulfide bonding. This prediction must be taken with caution as homology modeling using *Phyre*^2^ [92] does not yield similar 3-D prediction results. Nevertheless, both prediction tools indicate that the secondary structure mostly consists of β-strands. Propeller proteins with five to eight blades are the most prevalent, with the six- and seven-bladed propellers displaying the highest degree of functional variation capable of mediating ligand binding, catalysis, or protein–protein interactions.

## 4. The Perplexing Roles of Vacuolar Targeting Signals (PEXELs and TEXELs) and Their Licensing Proteases in Apicomplexan Effector Protein Export

**Figure 9:**
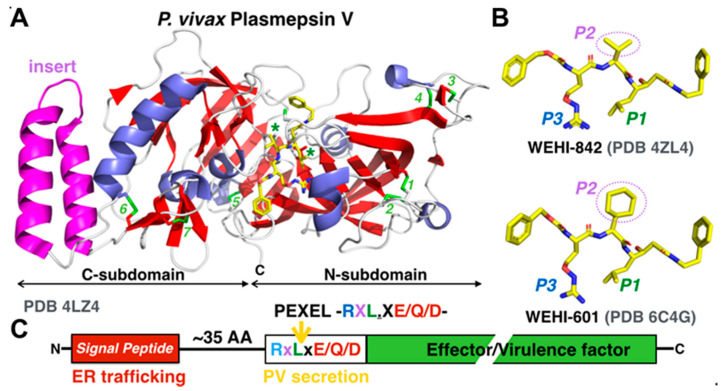
Inhibitors of the licensing aspartic protease plasmepsin V mimic the PEXEL vacuolar export signal. (**A**) Crystal structure of *P. vivax* plasmepsin V bound to a synthetic inhibitor. The active site cleft sits at the interface between the *N*- and *C*-terminal subdomains of the pepsin-like protease. The 2 conserved catalytic aspartates in motifs D_80_TGS and D_317_SGS/T (green asterisks) and conserved arrangement of 7 disulfide bonds are indicated. The helix-turn-helix insert (magenta) is characteristic of Plasmodium but not Toxoplasma. (**B**) Structures of two synthetic inhibitors of PlmV. The two inhibitors (WEHI-842 and WEHI-601) [95,96] differ by the chemical group filling up the *S2* protease active site pocket (P2 position of the inhibitor) and are shown in their protease-bound conformations. Both are potent inhibitors of malarial protein secretion. The inhibitors mimic the *RXL* motif of the *RXL*XE/Q/D PEXEL consensus. (**C**) Schematic organization of a PEXEL-dependent effector protein. The *N*-terminal signal sequence determining trafficking through the ER–Golgi–vesicular secretory network is separated from the downstream PEXEL motif by ~35 residues.

The discovery of vacuolar-targeting signals in Plasmodium and Toxoplasma and their proteolytic licensing by aspartic acid proteases not only constituted a major advance in our basic understanding of apicomplexan protein trafficking [93], but it also opened new avenues for the development of anti-parasitic compounds. A large subset of malarial effectors contain a PEXEL targeting signal, a pentapeptidic motif—RXLXE/Q/D—that is processed by the ER-resident aspartic protease plasmepsin V (PlmV). PEXELs are located around ~35 residues downstream of the *N*-terminal signal sequences. Following signal sequence processing by signal peptidase complexes [19], PEXEL cleavage occurs after the conserved leucine residue. *N*-acetylation of PEXEL-processed termini by a yet uncharacterized enzyme is common yet not systematic. Plasmepsins form a diverse group of pepsin-like aspartic proteases in the malaria parasite. Ten plasmepsins have been identified; they are involved in various aspects of parasitic life, such as hemoglobin catabolism (PlmI-IV), egress and invasion (PlmIX and X), and effector export (PlmV) [94]. The crystal structures of *P. vivax* PlmV bound to potent synthetic inhibitors have revealed a plant-like fold and a malaria-specific insertion motif important for the cleavage of exported effectors [95] (Figure 9, Figure S6).

A growing and significant number of exported proteins lacking a PEXEL motif, PEXEL-negative proteins (PNEPs), have been identified and are not substrates for PlmV [97]. Most PNEPs contain one TM helix but lack a canonical signal peptide; PNEP TMs and some *N*-terminal sequence features seem to provide the necessary targeting elements for proper export, although the molecular mechanisms remain mysterious [98,99]. Remarkably, PTEX is required for the export of both protein classes, PEXEL-containing effectors and PNEPs, highlighting its importance as the central nexus in effector protein trafficking [29,30].

In Toxoplasma, a vacuolar trafficking signal has also been identified but only for a limited subset of effectors, such as proteins GRA16 and GRA24. This TEXEL motif (Toxoplasma export element) consists of the simpler consensus [-RRL-] tripeptide [16,100]. However, unlike PEXELs, TEXELs do not appear to be spatially restricted to the *N*-terminus of the protein and do not necessary target GRA proteins for export beyond the PVM, and most Toxoplasma effectors are TEXEL-negative proteins (TENEPs) [101]. TEXEL-containing proteins are cleaved after the RRL motif by the Golgi-resident aspartic acid protease Asp5, the Toxoplasma orthologue of plasmepsin V (Figure S6), whose activity is necessary for the translocation of effector proteins whether or not they harbor a TEXEL motif [17,102]. The apparent lack of spatial restriction of TEXELs, by comparison with PEXELs, is somehow perplexing and it has led to the hypothesis that cleavage by Asp5 might expose another and more cryptic cue to trigger export.

While some of the modalities for unfolded cargo binding might be explained by comparing the PTEX HSP101 structure with its bacterial ClpB and yeast HSP104 orthologues, the molecular mechanisms of cargo targeting and delivery to the PTEX machinery itself in general and more specifically to HSP101 remain unknown. For PEXEL-containing proteins, it is unclear if specific recognition of a processed/*N*-acetylated PEXEL motif (by the NTD in HSP101?) is required for the physical delivery of licensed cargo to the HSP101 hexamer assembly in PTEX. The intervention of specific adaptor proteins is well established for the ClpA and ClpC class I AAA+ chaperones’ proteins that function in association with ClpP proteases. However, there is no indication that this is the case for the malarial vacuolar secretion pathway. For most of their protein remodeling activities, ClpB and HSP104 function in collaboration with DnaK or HSP70 chaperones, respectively [103]. In Plasmodium, the HSP70 system is expanded, likely participating in cargo delivery to the HSP101 chaperone for subsequent translocation and also assisting in cargo refolding with its HSP40 co-chaperones in the red blood cell cytoplasm after translocation [104,105]. By analogy with the ClpB/HSP104 systems, direct molecular interactions between these DnaK/HSP70 chaperones and the middle domain insertion of HSP101 could direct the delivery and engagement of substrates into this ATPase-dependent translocon.

## 5. Secretion Across the Parasitophorous Vacuole in Other Apicomplexans: Toxoplasma and Other Coccidia

PTEX is the first effector apicomplexan protein vacuolar secretion system that has been extensively characterized functionally and structurally. *Toxoplasma gondii* is another apicomplexan parasite dangerous to humans. Contrary to Plasmodium that targets hepatic and erythrocytic cells in humans, Toxoplasma is a quite promiscuous parasite, capable of infecting most nucleated cell types. Despite this difference in host cell tropism, Toxoplasma also hijacks its host cells and hides within a parasitophorous vacuole derived from the host cell upon invasion [8].

**Figure 10:**
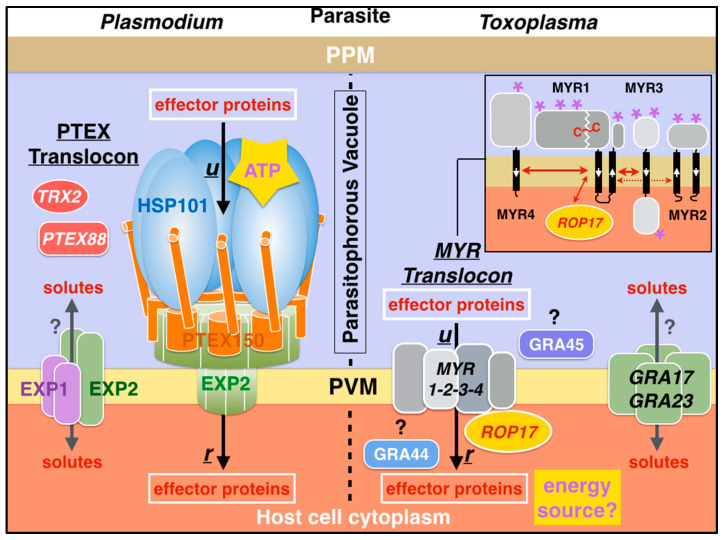
Comparison of the vacuolar translocation and transport pathways in the apicomplexans Plasmodium and Toxoplasma. Effector protein translocation is mediated by the PTEX in Plasmodium and the MYR complex in Toxoplasma. ***u*** and ***r*** indicate unfolding and refolding steps prior to and after translocation. PTEX subunit EXP2, in association with another vacuolar membrane protein EXP1, is involved in the transport of small molecules across the membrane; this activity seems independent from the protein secretion activity [36]. The rhoptry kinase ROP17 phosphorylates one or several MYR proteins and is required for effector translocation. Contrary to PTEX, MYR does not seem to use an AAA+ unfoldase. The inset describes the possible topologies of insertion of the four MYR proteins in the vacuolar membrane based on biochemical data and structural predictions of transmembrane domains (black thick lines with white arrows pointing towards the *C*-termini). MYR1 and MYR3 form a stable interaction. Red double-headed red arrows highlight strong interactions between MYR1 and proteins MYR3 and MYR4 while interaction with MYR2 is transient. The two fragments generated upon proteolytic cleavage of the TEXEL in MYR1 are disulfide linked. Purple asterisks indicate putative serine phosphorylation sites [106,108]. Three additional proteins MYR4, GRA44, and GRA45 complete the MYR translocon [109]. The insertion topology of MYR proteins and cellular localization of GRA44 (vacuolar lumen vs. host cytoplasm) are not yet elucidated. GRA45 has been localized to the vacuole lumen [110].

Initial attempts to characterize the components responsible for vacuolar export in Toxoplasma revealed that a vacuolar membrane-associated protein complex composed of the three proteins MYR1, MYR2, and MYR3 is essential for GRA effector translocation across the parasitophorous vacuole membrane [8,10,106]. Despite the identification of an extended panoply of AAA+ Clp-like proteins in Toxoplasma [107], no orthologue of the vacuolar Plasmodium HSP101/ClpB2 unfoldase has been identified in this apicomplexan; nevertheless, protein unfolding is also a necessary step for vacuolar export of Toxoplasma effectors [10]. A fourth protein, the secreted parasitic kinase ROP17, is also required for translocation [108]; ROP17 is likely to phosphorylate one or more of the components of the MYR translocon and thus potentially energize translocation. Recently, three additional proteins, MYR4, GRA44, and, GRA45, were also identified as essential for the export of GRA effectors in Toxoplasma [109]. GRA44 is a putative phosphatase, while GRA45, predicted to contain a small heat shock protein domain and a transthyretin-like domain, is critical in preventing other GRA effectors from aggregating and thus functions as a chaperone (Wang Y. et al., 2019. bioRxiv doi:10.1101/867705) (Figure 10, Figure S7).

As mentioned before, Asp5 activity is necessary for translocation of all exported Toxoplasma GRA effectors whether or not they contain a cleavable TEXEL motif. Furthermore, some MYR components, such as MYR1, MYR4, GRA44, and GRA45 but not MYR2 or MYR3, contain TEXEL RRL motifs (Figure S7). This requirement might indicate that at least one or perhaps several components of the translocation machinery (e.g., a chaperone and/or a transmembrane component with the exception of MYR1) must be processed by Asp5 in order for effectors to be exported whether they contain a TEXEL or not.

All these proteins are necessary for effector export in Toxoplasma, yet very little is known about their structure and organization into a functional vacuolar translocation complex. As in the case of Plasmodium EXP2, PTEX150, and PTEX88, surveys of protein sequence and protein structure databases combined with secondary structure predictions fail to reliably identify any known functional or structural homologues. For example, the *C*-terminus part of MYR1, where two TMH are predicted, is hypothesized to bear some resemblance with protein TatC, one of the three components of the twin-arginine translocon system (Tat), an Sec-independent periplasmic translocase capable of translocating folded proteins, present in Bacteria, Archea but also plastids and mitochondria [106]. The sequence homology is, however, weak and such predictions should be taken with caution, as they can be misleading as exemplified by the case of *Pf*-EXP2, erroneously predicted to resemble a hemolysin E bacterial toxin. Overall, the structural basis and energetics of vacuolar secretion in Toxoplasma remain largely unknown.

## 6. Parasitic Vacuolar Secretion Pathways as Drug Targets

Current strategies to eradicate malaria include the development of vaccines [111] but also strategies targeting the mosquito reservoir population either by the use of insecticide-treated mosquito nets or more complex strategies using self-propagating CRISPR-based gene drive [112] to ‘control’ mosquito populations. Prophylaxis and medical treatment also include the use of a powerful arsenal of anti-malarial drug combinations. Efforts to control malaria are, however, threatened by the emergence of widespread drug resistance and the relative overall poor chemical diversity of available drugs; this is further aggravated by the improper use of antimalarial drugs at hand and the existence of counterfeited drugs. Identification and molecular characterization of biological pathways unique and essential to the parasite are crucial to identify novel drug targets.

Knowledge of atomic or near-atomic resolution structures of isolated PTEX components or PTEX core complexes opens new avenues for the rational design of much needed novel classes of antimalarial compounds: Inhibitors of effector protein trafficking and secretion. Several steps along the complex journey of effector proteins through the parasitic vacuolar secretion pathway can be targeted [113]. Inhibition of cargo licensing by the ER-associated aspartic protease plasmepsin V is a conventional approach that is already explored [95,96,114]; it benefits from a large body of knowledge on the structure of protease active sites and their associated medicinal chemistry [114] (Figure 9, Figure S6). A similar strategy could be applied to the essential TEXEL-licensing protease Asp5 in Toxoplasma.

Following the same rationale, the purely enzymatic components of PTEX, namely HSP101 and TRX2, could be targets for inhibitor screening and design. Although HSP101 is a member of the large and ubiquitous AAA+ protein superfamily, specific inhibitors of distinct AAA+ proteins have been successfully designed in other unrelated systems [115,116]. A similar observation can be made for TRX2 [85]. Plasmodium multiplies and differentiates rapidly in various intra- and extracellular environments and in two different hosts (i.e., mosquito and human). Due to its life cycle, Plasmodium is exposed to high levels of reactive oxygen species resulting from its high metabolic rate, hemoglobin catabolism, and host immune response. In response to these challenges, Plasmodium has evolved expanded antioxidant defense mechanisms [117].

Novel approaches should target protein components or structural features really unique to this secretion system. Assembly of the PTEX core and blockade of the PTEX150/EXP2 channel by itself appear as targets of choice. Indeed, they are only very few small molecule inhibitors known to directly bind to and specifically block a translocon: Natural products, such as cyclic heptadepsipeptides [118,119] and decadepsipetides [120,121] (i.e., cotransin, decatransin, and apratoxin), were shown to specifically inhibit general protein translocation by binding to and interfering with the lateral gate of the central Sec61α channel regardless of ribosomal activity. By analogy, if such molecules can be developed in the unique case of PTEX, it is likely that the combination of several compounds targeting different steps might prove particularly powerful antimalarial therapies.

All MYR translocon proteins (MYR1-4, ROP17, GRA44, and GRA45) identified in *Toxoplasma gondii* have orthologues in other apicomplexan parasites, such as *Neospora caninum* and *Hammondia hammondi*, although the levels of sequence divergence suggest rapid evolutionary pressure [10,106,109]. However, no homologues of MYR proteins have been identified in Sarcocystis (also an Eucoccidioridia) or the more distantly related Plasmodium; thus, MYR proteins appear somehow restricted to a subset of Coccidia, perhaps as an adaptation to the rapid evolution of their specific effectors and life cycle (host cell). Apicomplexan genera Neospora and Hammondia cause a different type of parasitosis, broadly classified as coccidiosis, which can be dangerous to humans but also pets (cats and dogs), livestock, and poultry. Because of their impact not only on human but also animal health, there is practical interest in understanding and targeting MYR-based effector protein translocation in a wide range of apicomplexans to develop potential novel anti-parasitic drugs targeting the secretory pathway in Coccidia.

## 7. Conclusions

The existence of a parasitophorous vacuole in apicomplexans raises a flurry of questions regarding the molecular mechanisms of protein targeting, insertion, and secretion. We do not yet understand in what state(s) proteins destined for insertion inside the PVM (such as EXP1, EXP2, and many other single-pass PV membrane proteins) or translocation across the PVM (effector proteins) are delivered to the membrane or PTEX, respectively. Proteins are maintained in an unfolded state presumably associated with chaperones but the molecular machineries involved in these steps remain largely unknown. It has been proposed that free HSP101 hexamers in the PV might be able to chaperone substrates while they are exiting the parasitic secretory pathway and transiting at the interface between the parasite plasma membrane and the PV lumen itself. Once translocated, effector proteins will eventually require refolding; again, host-derived and/or parasitic chaperones (such as HSP70-x and HSP40) are likely to be involved.

Structural biology combined with molecular genetics and cellular biology approaches have rapidly expanded our knowledge of the composition, structure, and molecular mechanisms governing vacuolar secretion in two apicomplexans, Plasmodium and Toxoplasma. So far, there is no evidence that MYR-like proteins are present in Plasmodium and, conversely, that a system similar to PTEX exists in Toxoplasma. Effector protein vacuolar secretion is an essential process shared by these pathogenic protozoa, and while the thermodynamics of translocation across a membrane bilayer are universal, it seems that the molecular machineries evolved to fulfill this function are remarkably different. This most likely reflects the incredible diversity of species, parasitic life cycles (i.e., hosts, vectors, and host cell specificity), and differences in selective pressures.

While the structure of PTEX reveals remarkable and unexpected features, such as the structures of PTEX150 and of a novel pore-forming and “toxin-like” membrane protein EXP2, it also shares some analogies with some bacterial secretion systems (TSS) powered by AAA+ proteins and the injection of virulence factors into their host/target cells through sophisticated molecular syringes. Because of its lack of obvious structural homologues, the MYR machinery seems quite different and promises to be particularly original. The structural basis for MYR-mediated vacuolar protein export in *Toxoplasma* and other Coccidia remains mysterious and will necessitate further studies as they are likely to reveal novel membrane protein complex architectures and mechanisms of translocation.

## Figures and Tables

**Table 1:** Protein Data Bank identifiers for PTEX protein structures.

PTEX subunit	HSP101	PTEX150	EXP2	TRX2	PTEX88
PDB ID	6E10	6E10	6E10		NA
	6E11	6E11	6E11		
	4IOD			3UL3	
	4IRF			4O32	

Identifiers in red and blue correspond to cryo-EM and crystallographic structures, respectively.

**Table 2:** Amino acid usage in PTEX subunits.

PTEX subunit	HSP101	PTEX150	EXP2	TRX2	PTEX88
Number of Residues	906	993	287	157	777
% composition ^a^					
% Asn	6	17	5	7	13
% Asp	5	13	12	6	6
% Glu	8	10	8	2	5
% Lys	12	10	11	13	10
structural coverage ^b^	100%	20%	78%	65–75%	NA

^a^ % of a given amino acid corresponds to the percentage composition of the residue (signal sequences have been excluded from the calculation). ^b^ structural coverage corresponds to the percentage of expected protein sequence that is modeled in the final X-ray diffraction or cryo-EM density maps. Although the total number of residues is indicated line 2 of the table, % coverage is calculated using the protein sequence after signal sequence processing.

## Supplementary Materials

The following are available online at https://www.mdpi.com/2076-2607/8/6/865/s1, Figure S1: Protein sequence alignment of EXP2 from 12 *Plasmodium* species [122,123]; Figure S2: Protein sequence alignment for the core segment of PTEX150 from 10 *Plasmodium* species; Figure S3: Splayed surface representation of the EXP2/PTEX150 tetradecameric pore assembly; Figure S4: Comparison of three protein-conducting pores; Figure S5: Protein sequence alignment of the catalytic domains of AAA+ protein unfoldase/disaggregases *Pf*-HSP101, *Ec*-ClpB, and *Sc*-HSP104; Figure S6, Protein sequence alignment of the catalytic domains from aspartic proteases *P. vivax* Plasmepsin V, *P. falciparum* Plasmepsin V and *T. gondii* Aspartic Protease 5 involved in the proteolytic licensing of PEXEL and TEXEL motifs, respectively; Figure S7: Sequences and properties of *Toxoplasma gondii* MYR vacuolar membrane proteins.
