# Supplementary material for: Crossing the Vacuolar Rubicon: Structural Insights into Effector Protein Trafficking in Apicomplexan Parasites
Source: Microorganisms. 2020 Jun 8;8(6):865. doi: 10.3390/microorganisms8060865 (PMC7355975; doi:10.3390/microorganisms8060865)
Supplement: Supplementary file 1 [file microorganisms-08-00865-s001.pdf]

Supplementary Figure Legends and Supplementary Figures

Figure S1. ***Protein sequence alignment of EXP2 from 12 Plasmodium* species.** The secondary structure elements and domain boundaries as observed in the reference structure of *Pf*-EXP2 [26] are indicated on top of the alignment. The pair of strictly conserved cysteines engaged in a disulfide bridges is highlighted in green. Other highly conserved cysteines are highlighted in grey. Alignment performed in *Clustal-Ω* [122] and displayed with *ESPRIPT3.0* [123]. Although the predicted signal sequence cleavage site is between residues 20-22, the magenta arrow indicates the observed cleavage site after the first cysteine in *Pf*-EXP2. Sequences were retrieved from www.PlasmoDB.org. Human pathogens: *P.falciparum, P.vivax, P.malariae, P.ovale* – Monkey pathogens: *P.knowlesi, P.cynomolgi, P.reichenowi* – Rodent pathogens: *P.berghei, P.yoelii, P.chabaudi* – Bird pathogens: *P.relictum, P.gallinaceum*. The species infecting monkeys have also been found in humans.

Figure S2. ***Protein sequence alignment for the core segment of PTEX150 from 10 Plasmodium species.*** (**A**) The secondary structure elements and domain boundaries as observed in the reference structure of *Pf*-PTEX150 [26] are indicated on top of the alignment. Alignment performed in *Clustal-Ω* and displayed with *ESPRIPT3.0*. Human pathogens: *P.falciparum, P.vivax, P.malariae, P.ovale* – Monkey pathogens: *P.knowlesi, P.cynomolgi, P.reichenowi* – Rodent pathogens: *P.berghei, P.yoelii, P.chabaudi*. The species infecting monkeys have also been found in humans. Sequences were retrieved from www.PlasmoDB.org. (**B**) Matrix displaying the percentage of sequence identity.

Figure S3. ***Splayed surface representation of the EXP2/PTEX150 tetradecameric pore assembly.*** The EXP2 heptamer and EXP2/PTEX150 tetradecamer are shown in surface representation where three EXP2 (1, 2 and 7) and three PTEX150 (1′, 6′ and 7′) subunits have been rendered transparent to show the geometry of the protein conducting path and the intricacy of the association between the soluble adaptor protein PTEX150 and the membrane protein EXP2. Subunits are labeled and colored as in Figures 3 and 4. A model helix (P) indicates the axis of the central pore along the protein-conducting path.

Figure S4. ***Comparison of three protein-conducting pores.*** Side, top and bottom views of **(A)** the anthrax protective antigen (PDB 3J9C) [47], a C7-symmetric β pore-forming translocon with a ring of residues (ϕ-clamp, magenta) functioning as an acidic pH-sensing gate, **(B)** the rigid EXP2/PTEX150 tetradecamer and **(C)** the Sec61/SecY universal translocon (PDB 3MP7) [50]. Transmembrane regions (β-strands or α-helices) are colored in green or cyan, soluble regions are colored in grey. **(A)** and **(B)** are on the same scale, **(C)** is 2.4X the scale of **(A)** and **(B)**. For Sec61/SecY, the hydrophobic plug (yellow) and ring (magenta) regions seal the pore and prevent leakage of water and ions in the resting state or during translocation. The lateral gate provides an exit portal (dark dots) to partition transmembrane helices from the pore into the lipid bilayer.

Figure S5. ***Protein sequence alignment of the catalytic domains of AAA+ protein unfoldase/disaggregases Pf-HSP101, Ec-ClpB, and Sc-HSP104.*** For the sake of clarity, the N-terminal domains have been omitted. The secondary structure elements and domain boundaries as observed in the reference structure of *Pf*-HSP101 [26] are indicated on top of the alignment. Alignment performed in *Clustal-W* and displayed with *ESPRIPT3.0*. Nucleotide-Binding Domains (NBD) I and II, C-Terminal Domains (CTD) and middle domain (MD) are indicated. Walker A (-GxxxxG**K**T/S- x being any residue) and Walker B (-hhhh**DE**- h being any hydrophobic residue) motifs bearing the catalytic residues are indicated together with the pore loop (PL) tyrosines (Y280 and Y686) interacting with the polypeptidic backbone of denatured substrates. The *trans*-acting Arginine fingers (R360/R361 and R800) and the *cis*-acting Arginine finger (R859) are involved in *inter* and *intra*- protomer transition state stabilization, respectively.

Figure S6. ***Protein sequence alignment of the catalytic domains from aspartic proteases P. vivax Plasmepsin V, P. falciparum Plasmepsin V and T. gondii Aspartic Protease 5 involved in the proteolytic licensing of PEXEL and TEXEL motifs, respectively.*** Sequence alignment of the conserved catalytic domains of *Pv*-PlmV, *Pf*-PlmV and *Tg*-Asp5. Alignment performed in *Clustal-W* and displayed with *ESPRIPT3.0*. The secondary structure elements and domain boundaries as observed in the reference structure of *Pv*-PlmV are indicated on top of the alignment [95]. Catalytic aspartates in conserved motifs **D_{80}**TGS and **D_{317}**SGS/T are underlined in green. The 7 conserved disulfide bonds are numbered and colored accordingly; the only non-bridged cysteine (*C**) is not conserved between *Toxoplasma* and *Plasmodium*. A helix-turn-helix insertion also characteristic of *Plasmodium* is colored in magenta. Predicted C-terminal trans-membrane helices (TMH) responsible for ER- (in PlmV) or Golgi- (in Asp5) anchoring are indicated. *Pv*-PlmV has 540 residues, *Pf*-PlmV has 590 residues and *Tg*-Asp5 has 1012 residues. Sequences were retrieved from *www.PlasmoDB.org* and *www.ToxoDB.org*.

Figure S7. ***Sequences and properties of Toxoplasma gondii MYR vacuolar membrane proteins.*** Predicted signal sequences and trans-membrane helices are boxed in grey and yellow, respectively. Cysteines are boxed in red. Serine-phosphorylation sites are boxed in cyan; MYR1: S175, S227, S246 and S787 – MYR2: S157 and S162 – MYR3: S41, S52 and S115 – MYR4: S724. TEXEL vacuolar trafficking signals (-**RRL**-) processed by aspartic protease *Tg*-Asp5 are present in MYR1 and MYR4. For the MYR4 sequence, numbering adheres to the results from Cygan et *al.* resulting in a discrepancy with the sequence curated in *www.ToxoDB.org* where the encoded protein has an additional 60 residues at its N-terminus [109]. For this shorter protein, this results in the high-probability prediction of a signal sequence.

Figure S1: *Plasmodium spp.* Exported Protein-2 (EXP2) sequence alignments

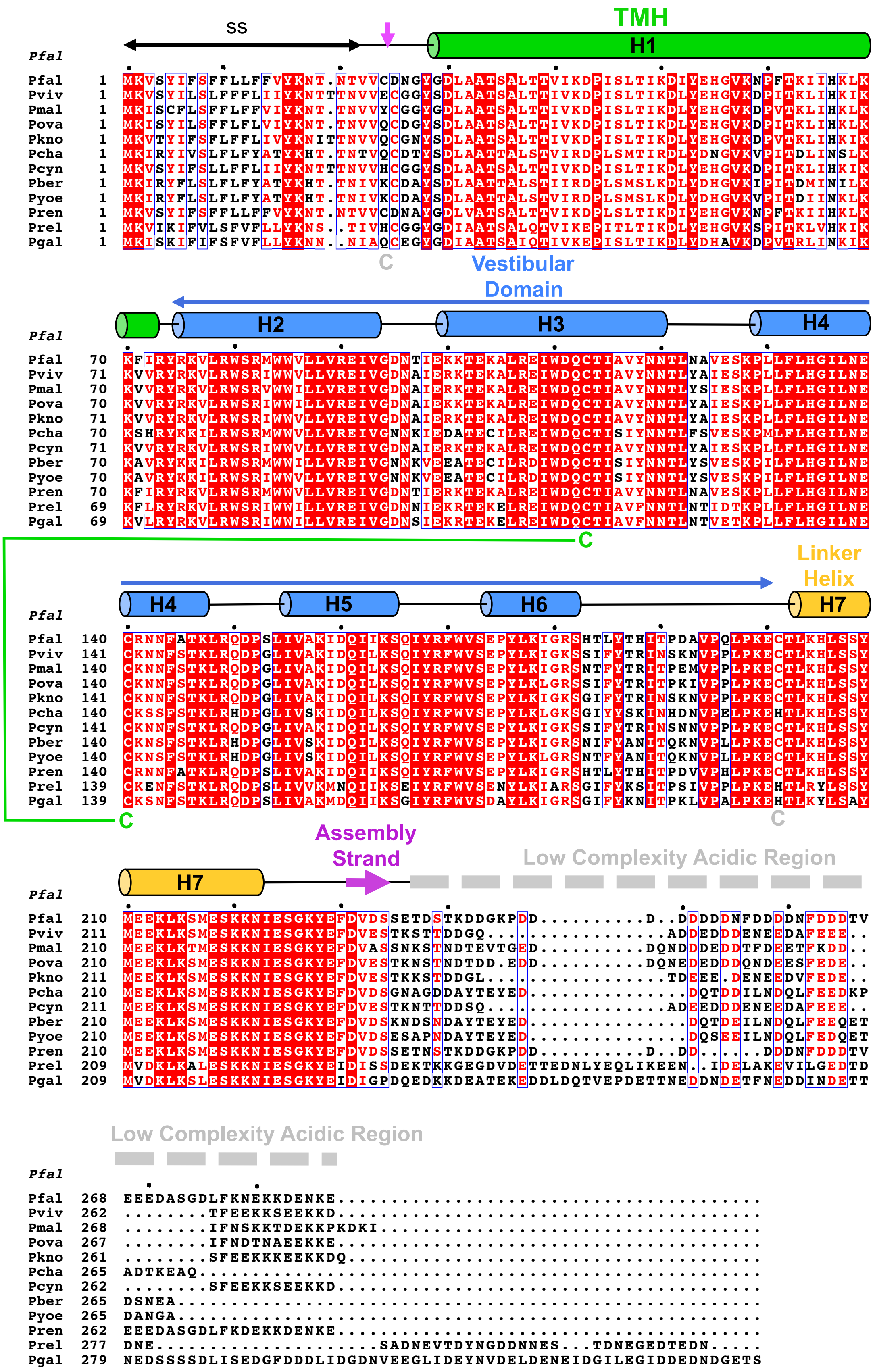

Figure S2: *Plasmodium spp.* structured core PTEX150 sequence alignments

A

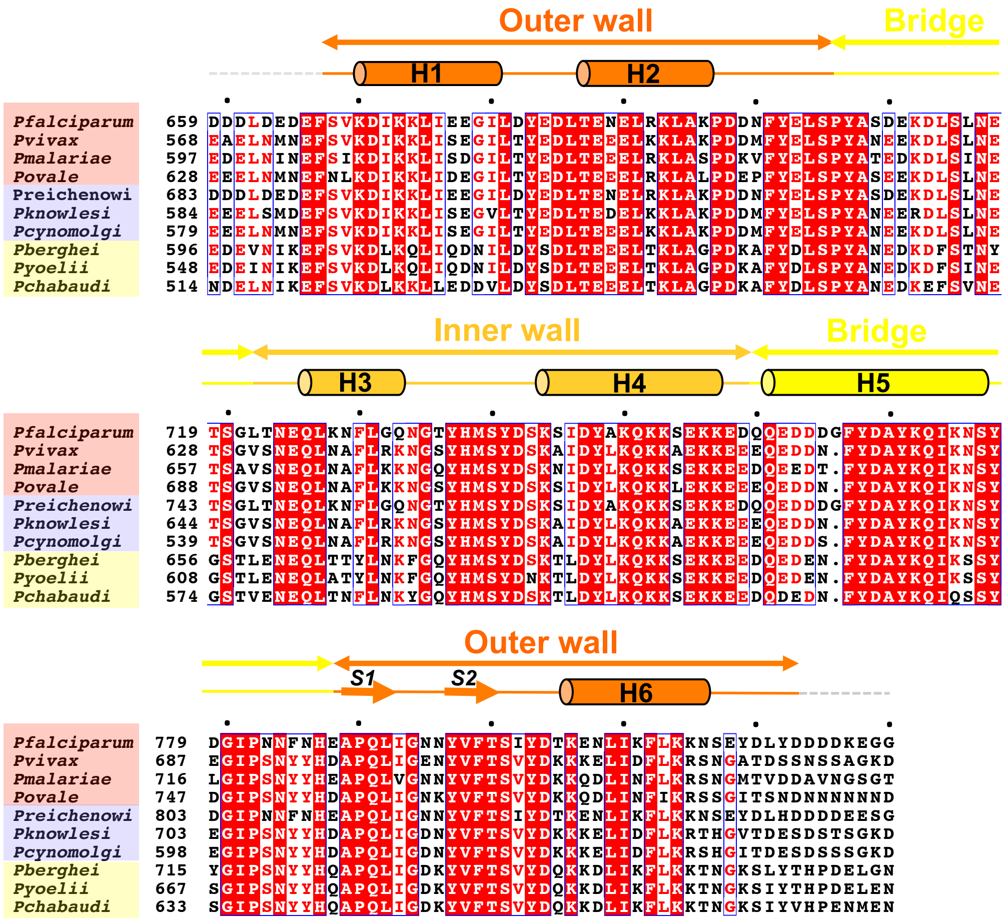

B

Human pathogens
P.falciparum (gene PF3D7_1436300)
P.vivax (gene PVX_084720)
P.malariae (genePmUG01_13024000)
P.ovale (gene PocGH01-13024000)

Monkey pathogens
P.reichenowi (gene PRG01_1436300)
P.knowlesi (gene PKNH_0422900)
P.cynomolgi (gene PCYB_132260)

Rodent pathogens
P.berghei (gene PBANKA_1008500)
P.yoelii (gene PY17X_1010100)
P.chabaudi (gene PCHAS_1009400)

P.falciparum	**100.00**									
P.vivax	69.01	**100.00**								
P.malariae	67.84	78.36	**100.00**							
P.ovale	69.59	83.04	80.12	**100.00**						
P.reichenowi	**98.26**	69.01	67.25	69.59	**100.00**					
P.knowlesi	69.01	**91.81**	74.27	79.53	69.01	**100.00**				
P.cynomolgi	69.59	**95.32**	77.78	83.04	69.59	94.74	**100.00**			
P.berghei	62.57	66.67	69.01	64.33	61.99	64.91	66.08	**100.00**		
P.yoelii	61.40	66.67	69.01	64.33	61.40	64.91	66.08	95.32	**100.00**	
P.chabaudi	60.82	66.08	69.01	64.33	60.23	65.50	65.50	85.38	86.55	**100.00**

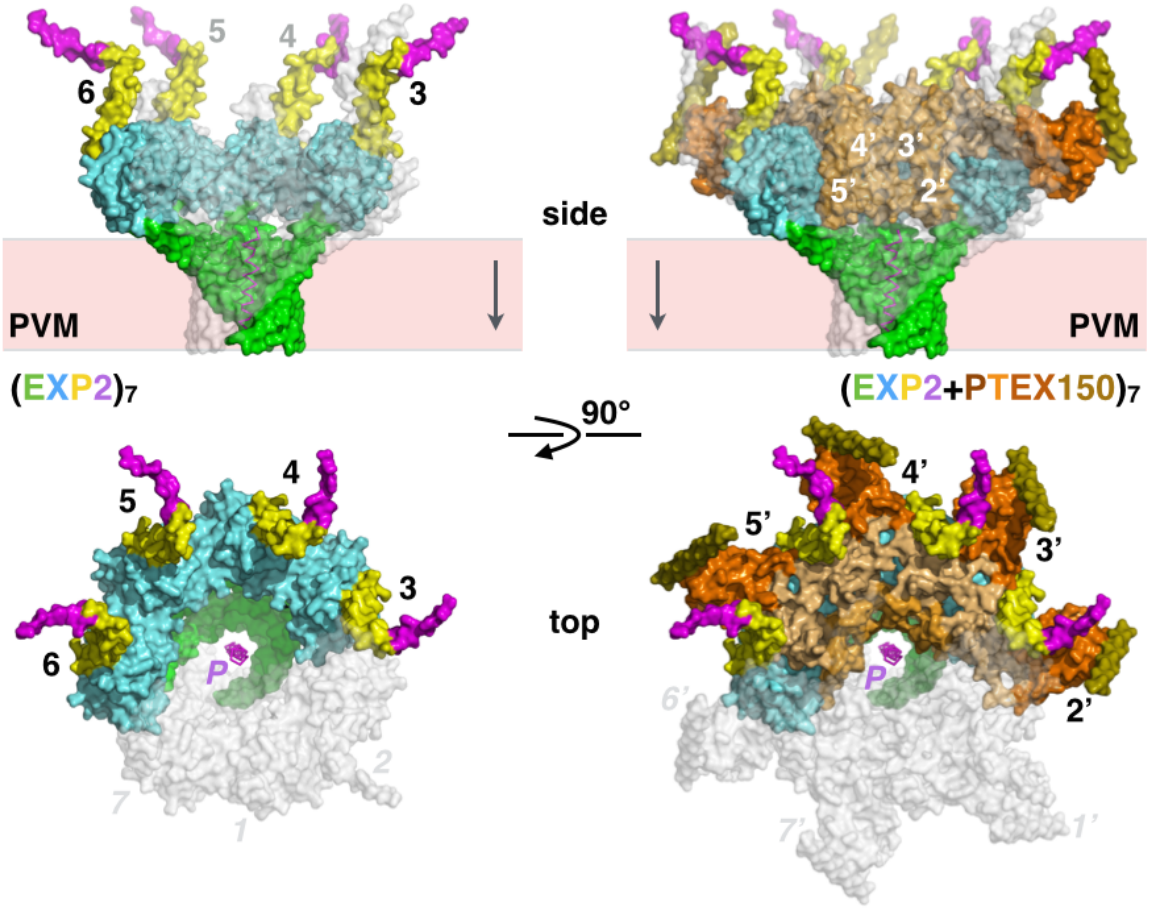

Figure S3. *Splayed surface representation of the EXP2/PTEX150 tetradecameric pore assembly.* The EXP2 heptamer and EXP2/PTEX150 tetradecamer are shown in surface representation where three EXP2 (1, 2 and 7) and three PTEX150 (1', 6' and 7') subunits have been rendered transparent to show the geometry of the protein conducting path and the intricacy of the association between the soluble adaptor protein PTEX150 and the membrane protein EXP2. Subunits are labeled and colored as in Figures 3 and 4. A model helix (P) indicates the axis of the central pore along the protein-conducting path.

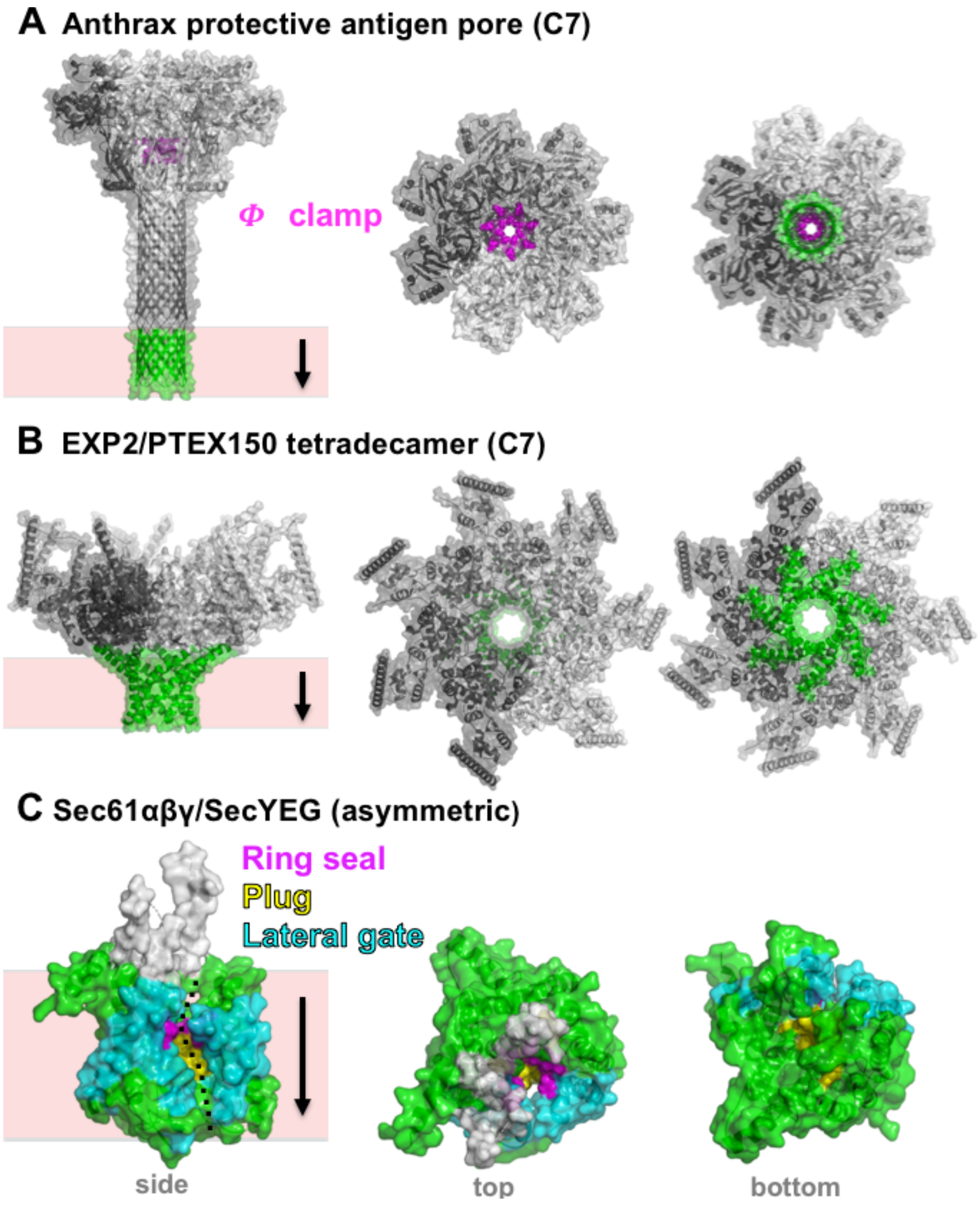

Figure S4. ***Comparison of three protein-conducting pores.*** Side, top and bottom views of **(A)** the anthrax protective antigen (PDB 3J9C) [44], a C7-symmetric β pore-forming translocon with a ring of residues (ϕ-clamp, magenta) functioning as an acidic pH-sensing gate, **(B)** the rigid EXP2/PTEX150 tetradecamer and **(C)** the Sec61/SecY universal translocon (PDB 3MP7) [47]. Transmembrane regions (β-strands or α-helices) are colored in green or cyan, soluble regions are colored in grey. **(A)** and **(B)** are on the same scale, **(C)** is 2.4X the scale of **(A)** and **(B)**. For Sec61/SecY, the hydrophobic plug (yellow) and ring (magenta) regions seal the pore and prevent leakage of water and ions in the resting state or during translocation. The lateral gate provides an exit portal (dark dots) to partition transmembrane helices from the pore into the lipid bilayer.

Figure S5: Sequence alignments of *Pf*-HSP101, *Ec*-ClpB and *Sc*-HSP104 Proteins

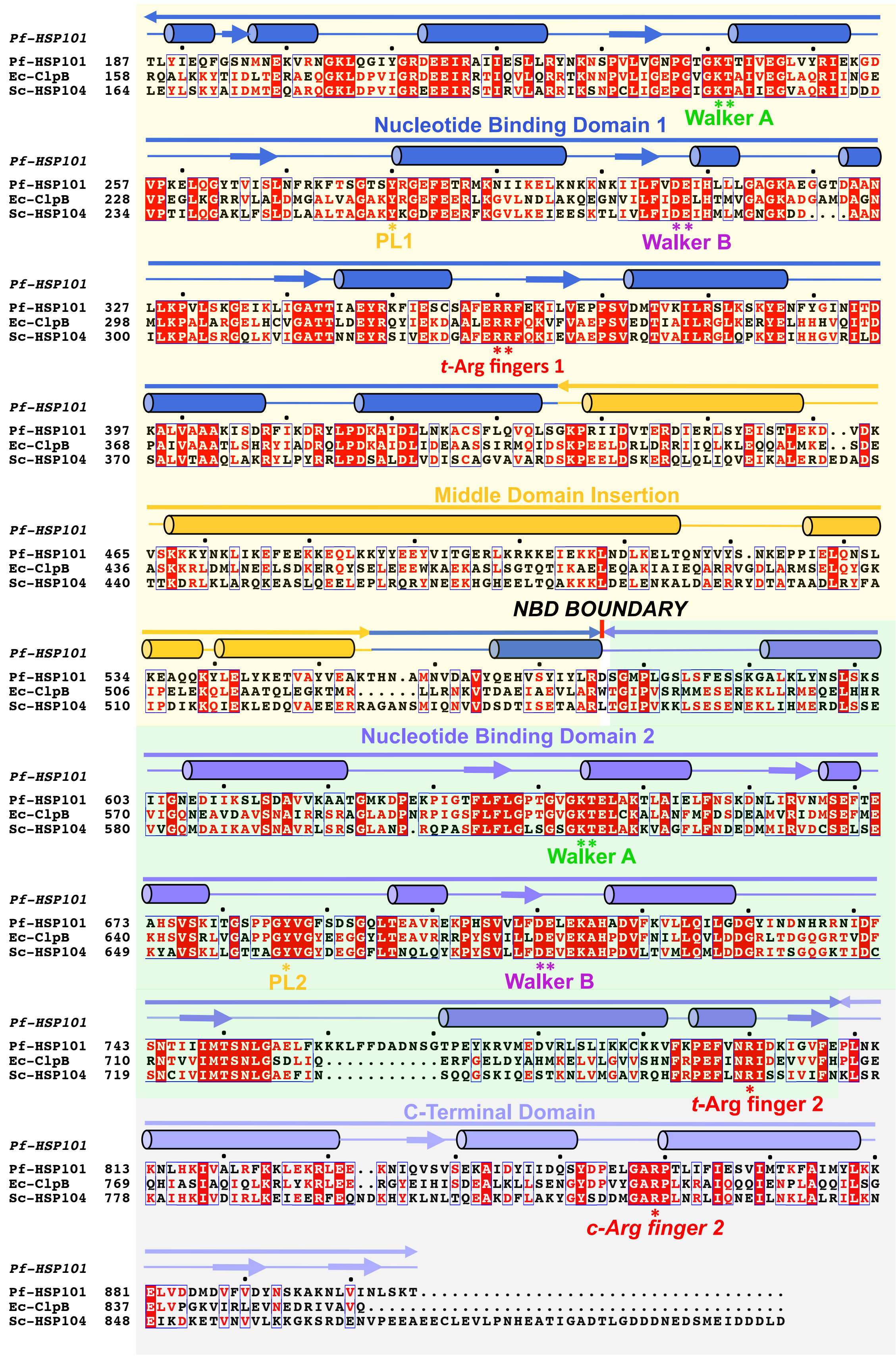

Figure S6: *Aspartic Proteases Processing Vacuolar Trafficking Signals*

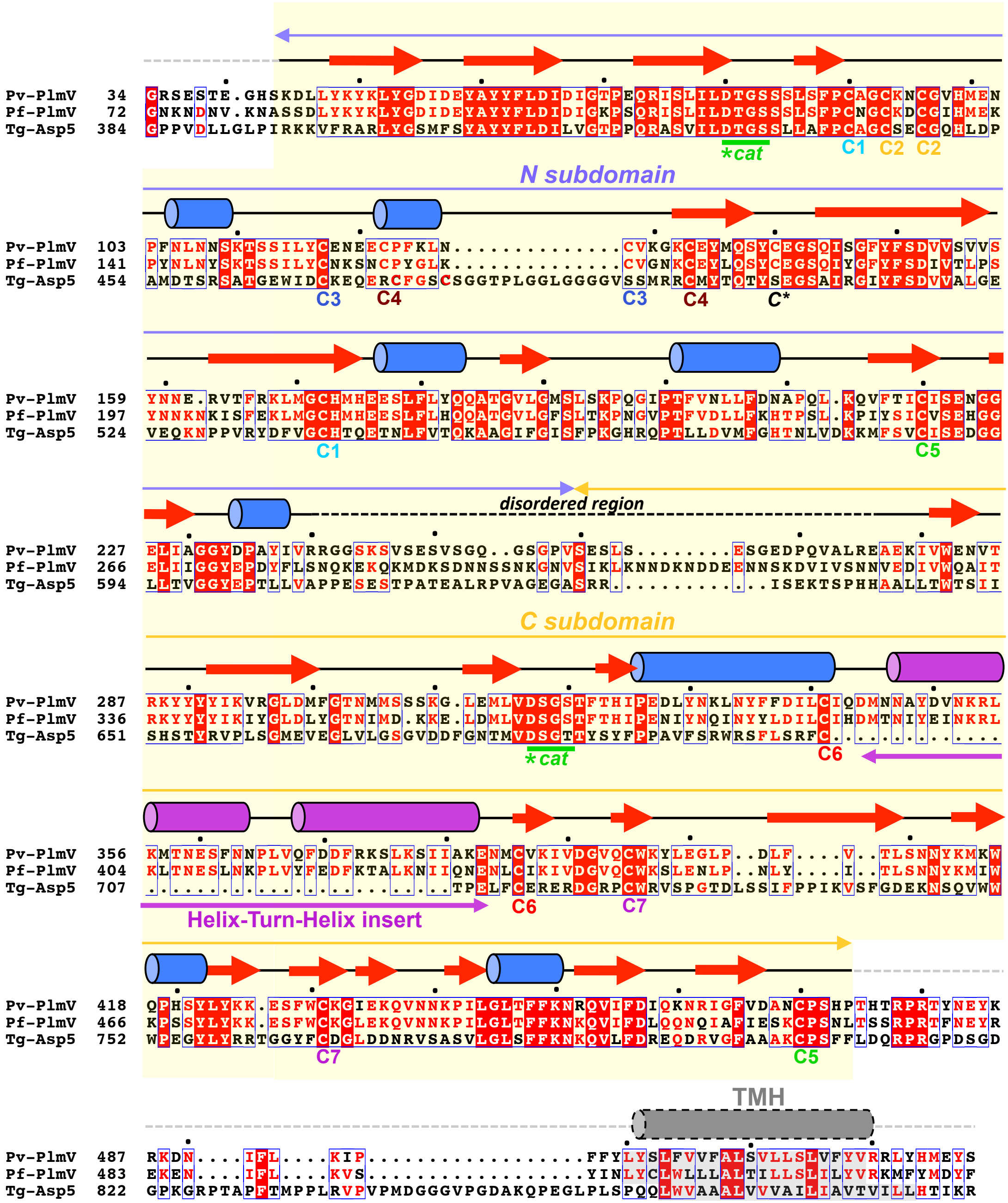

MYR1 (gene TGGT1_254470)

```
MRCLVALLCL VSSTALEGAE ATRAELLSVC LISLSLTGCF CCQPVAATDE SDLGNAFEQQ   60
RKDSESDESL NPEQEISGMP PQNRNEGKNS SPMTSDTSTF QGERGTDSPK GGENAEAGFL  120
GSLGVDAVLG ADGDDGYSDE DKAERGDPEF DMNSLDRQDQ ANPSEPRPSL PDEGSQGQHA  180
STGEPEVKSD NVKFISYAQK AEKNQPDGDF KGSESFSPSG FDYREGSPGV GDYRILNEGA  240
DSPTFSDHDY DDYDETSNGE LPLGRLRNKV QLTAAERYCG LKPKGPTVLY TPPGVRPDEL  300
EALDLFAVTD AGFFELLRDS HGDLVCPERR RGERVLFRNL RLKNSELVGD MVYSDPRSYR  360
HMTQVHPDGK ATKTPRAPTE QKTPPRREDR GSKERSKSVE NSGKKLSMWS RLLCKVSKRY  420
CNTKVTGEKG QDEDSGDPTT HKRQPRQIVA AEVPLREVFG VKPLEIFDWK VKELLYRLDR  480
EMGQEGNLDE RMPPYFAVPT EAAGRAPGVA QVPGSGSEGE TISHAGALQG DFQREPKGGD  540
AERRRESHET QNSAGAGRQA PASRTDAPVE AGQGDVRRLS EQAYNDNPEK APEQSGGALD  600
GGVDYPANDW LEAEYEDAPN ISIPDFLFSV PADRTHDDDE PNTMEQEATS VDLKVTVLCL  660
ENGAPPILIK EWPAFTNVVK PRKVLPQKPA VPRKSVAVWG SSFLAAVAAG MYAIKRLVNP  720
AALQYGSRYQ RLESFSLLGW LAVFVLGSAQ LAGLLTWNVR TAIHSSHLRH NLREELFADE  780
KAGTDGSAGS VYPTGFIFYY DRHFRQSHNS                                 810
```

MYR2 (gene TGGT1_270700)

```
MQSVADSFAV LIQFIAISEP PATVARTPQT RRRRGGRARL VRACWNASVF FLFWCLATWA   60
QDRWTADLFI VGEGADTNFP MVDQPGGPAD RDGVAFERQD SGVDSGSQEV GFDFENQGFG  120
ADLGGQDGGT SMRDGDQRTT HNGALSLKSP TGGDTQSEAD LSGESSSGHE APKQGEPPEQ  180
GDPPGQAVIP QAPVANSDFG YRGHSQVQAA PCVTRRCPYY KQPTEVIEEE NVMRKKPAVV  240
SRKRQASEWY YGDEETTTSK VLRGVGYGWM AFMVYAIIRH GMSNERTEPY YYEEQ       295
```

MYR3 (gene TGGT1_237230)

```
MGRRESLVGF ETGKLDSGVK HSQEDKASDD TAADALETND SSDNYGPRPP KSEDAARLRK   60
IYEQAHEGRK RQRKANFQAL LTSFGLVAYV AVSGFLFLRY ATPRSKRPQQ VGGGSDYGLH  120
DGYPVRHDNW YIRDNPNPPV LTMDQLFACE SERREGDRVG IESFTDCDGE AIGCFFAIRT  180
SECLERNQRD GRVYFEKKEL LDVDSSQAPP LCS                              223
```

MYR4 (gene TGGT1_211460)

```
MTTNRKGDVT VFRKERNQPS CTADVTMEGS SACGIPVAGQ NQRMAPLRND VRYRRCPRVA  -60
MTPRGGFERR ASALVPLSLL VITLLTKAEG KSSSNTEAKF ESHGAAQFSP LTVVNQPPAE   60
PLSAAVEHAV NNSNDPSGSH DPLLLNDGVD SDEGSRPSRQ EHEEADGWGQ FGSRHLADVT  120
QSGKQPQSED IQSGSQAGVA NHLSLAVGNS SSVKKEDVEQ GMSDETEFEP SHSDPVGKRH  180
PRRLRGEGAA AEAADAVRQL SVEPSSVSDP PSHPQFGVEQ ESEFVTGAEK RPPPTAVPVA  240
TTRAVQPVGI GVLSTTTLLA KRALEEVGSL GRGIGLYLYG VVFRGNLLLQ ANVLGKIKEN  300
IGVLEFVEEF QPQFAPSRRA IVDELTVWQD TLQRVVQQEQ AFVLQYQAYV SALREWSNRK  360
FHNDSGVHPT VSPVVDSQAS QAPPDSAPAT EQFSSETARD VDEQEVLEPS RNADITESRG  420
PGDLGLMAPG GDMAPSSVPS NAEQVNAYEF QSKPASSLVQ VNDTGDYSAT RFARQVVNEK  480
PPYTPHLRES SLPSAQVAVT GQPGDDEFVA HEGPETSEVL RASHSTDAGP DLVENPPEID  540
RTSSAIDTEM TDVDSVVVAS QGSEAVHNNS NADATQGPPF VVPRGVRGPL TTPPSMHASA  600
GIPPFFARKE DNDSADSPAK EVLPVSVAVD QHDEAIRESN GTKAPKEAGS SVLSTSHQNE  660
SSKGVALEDN SPGKSPSLPA VPVEGLPGTK AVGERIAAET EEAFSGSKGE TVHGHLSTPE  720
WVDSESDTER GLYGRQDSML LPPPDDVETL DLYLSLLQST FFKCGALLSR ALLADDSKEL  780
TWFYQEVLPS MSHLGRVSIA VVASQHRRGV RSAASAERHL QKIRSMLFHV TVVDDAFLHA  840
IYARDEPPTS ERLEASDSDE YRKPNARARS QKTKKDKVKV EGTRVGAAIE FAKAHLVKPW  900
VIGLLTFASL VGIDHYGYNR YY                                         922
```

Figure S7. ***Sequences and properties of Toxoplasma gondii MYR vacuolar membrane proteins.*** Predicted signal sequences and trans-membrane helices are boxed in grey and yellow, respectively. Cysteines are boxed in red. Serine-phosphorylation sites are boxed in cyan; MYR1: S175, S227, S246 and S787 – MYR2: S157 and S162 – MYR3: S41, S52 and S115 – MYR4: S724. TEXEL vacuolar trafficking signals (-RRL-) processed by aspartic protease *Tg*-Asp5 are present in MYR1 and MYR4. For the MYR4 sequence, numbering adheres to the results from Cygan et *al.* resulting in a discrepancy with the sequence curated in www.ToxoDB.org where the encoded protein has an additional 60 residues at its N-terminus [109]. For this shorter protein, this results in the high-probability prediction of a signal sequence.
